# Spleen plays a major role in DLL4-driven acute T-cell lymphoblastic leukemia

**DOI:** 10.7150/thno.48067

**Published:** 2021-01-01

**Authors:** Huizhong Xiong, Maicol Mancini, Michael Gobert, Shiqian Shen, Glaucia C. Furtado, Sergio A. Lira, Christopher N. Parkhurst, Veronique Garambois, Muriel Brengues, Carlos E. Tadokoro, Thomas Trimarchi, Gonzalo Gómez-López, Amartya Singh, Hossein Khiabanian, Sonia Minuzzo, Stefano Indraccolo, Camille Lobry, Iannis Aifantis, Daniel Herranz, Juan J. Lafaille, Antonio Maraver

**Affiliations:** 1 Kimmel Center for Biology and Medicine at the Skirball Institute, New York University School of Medicine, New York, NY 10016, USA.; 2 The Sackler Institute of Graduate Biomedical Sciences, New York University School of Medicine, New York, NY 10016, USA.; 3 Current address: Genentech, South San Francisco, CA, 94080.; 4 Institut de Recherche en Cancérologie de Montpellier (IRCM), Université de Montpellier, Institut Régional du Cancer de Montpellier (ICM), Montpellier, 34298, Cedex 5, France.; 5 Current address: Department of Anesthesia, Critical Care and Pain Medicine Massachusetts General Hospital, Boston, MA 02114, USA.; 6 Immunology Institute, Mount Sinai School of Medicine, New York, NY 10029, USA.; 7 Current address: Universidade Vila Velha, Vila Velha, 29102-920, Brazil.; 8 Department of Pathology, New York University School of Medicine, New York, NY 10016, USA.; 9 Bioinformatics unit. Spanish National Cancer Research Center (CNIO). Madrid, Spain.; 10 Rutgers Cancer Institute of New Jersey, Rutgers University, New Brunswick, NJ, USA.; 11 Department of Pathology and Laboratory Medicine, Rutgers Robert Wood Johnson Medical School, Rutgers University, New Brunswick, NJ, USA.; 12 Department of Surgery, Oncology and Gastroenterology, University of Padova, Padova, Italy.; 13 Veneto Institute of Oncology IOV - IRCCS, Padova, Italy.; 14 Institut de Recherche Saint Louis, INSERM U944/CNRS UMR7212, Paris, 75010, France.; 15 Howard Hughes Medical Institute.; 16 Department of Pharmacology, Robert Wood Johnson Medical School, Rutgers University, Piscataway, NJ, USA.

**Keywords:** DLL4, T-ALL, demcizumab, patient-derived xenografts, Notch pathway

## Abstract

The Notch pathway is highly active in almost all patients with T-cell acute lymphoblastic leukemia (T-ALL), but the implication of Notch ligands in T-ALL remains underexplored.

**Methods:** We used a genetic mouse model of Notch ligand delta like 4 (DLL4)-driven T-ALL and performed thymectomies and splenectomies in those animals. We also used several patient-derived T-ALL (PDTALL) models, including one with DLL4 expression on the membrane and we treated PDTALL cells *in vitro* and *in vivo* with demcizumab, a blocking antibody against human DLL4 currently being tested in clinical trials in patients with solid cancer.

**Results:** We show that surgical removal of the spleen abrogated T-ALL development in our preclinical DLL4-driven T-ALL mouse model. Mechanistically, we found that the spleen, and not the thymus, promoted the accumulation of circulating CD4^+^CD8^+^ T cells before T-ALL onset, suggesting that DLL4-driven T-ALL derives from these cells. Then, we identified a small subset of T-ALL patients showing higher levels of DLL4 expression. Moreover, in mice xenografted with a DLL4-positive PDTALL model, treatment with demcizumab had the same therapeutic effect as global Notch pathway inhibition using the potent γ-secretase inhibitor dibenzazepine. This result demonstrates that, in this PDTALL model, Notch pathway activity depends on DLL4 signaling, thus validating our preclinical mouse model.

**Conclusion:** DLL4 expression in human leukemic cells can be a source of Notch activity in T-ALL, and the spleen plays a major role in a genetic mouse model of DLL4-driven T-ALL.

## Introduction

The Notch pathway plays a major role in T-cell acute lymphoblastic leukemia (T-ALL). Indeed, more than 60% of patients harbor *NOTCH1* activating mutations and between 8 and 30% have inactivating mutations in *FBXW7*, a NOTCH1 negative regulator 1. In solid tumors, activating mutations in the Notch pathway are less frequent, but overexpression of Notch ligands in cancer cells has been reported as a mechanism to increase Notch pathway activity, for instance in breast and lung cancer 2, 3. However, to the best of our knowledge, Notch ligand expression has never been observed in patients with T-ALL, and their clinical relevance remains limited to seminal works that identified stromal Notch ligand delta like 4 (DLL4) as a source of Notch pathway hyperactivation in T-ALL 4, 5.

Conversely, using preclinical mouse models, we and others demonstrated that ectopic DLL4 expression in hematopoietic stem cells (HSCs) or T cells leads to spontaneous T-ALL development 6, 7 that is preceded by accumulation of non-clonal double-positive CD4^+^CD8^+^ cells in the circulation 6, 7. However, the tissue of origin of these peripheral CD4^+^CD8^+^ cells and their role in T-ALL were not known. Therefore, here, we used Tg8 mice, a genetic mouse model of T-ALL induced by DLL4 ectopic expression in T cells 6, and found that the spleen was the major peripheral lymphoid organ for extrathymic T cell development, with enrichment of all CD4^+^CD8^+^ cell precursors. Splenectomy inhibited peripheral CD4^+^CD8^+^ cell accumulation. Moreover, splenectomy, but not thymectomy inhibited DLL4-driven T-ALL development, suggesting that circulating CD4^+^CD8^+^ cells are at the origin of T-ALL in this mouse model.

Then, to determine the role of human DLL4 in T-ALL, we first analyzed *DLL4* mRNA expression in T-ALL samples and found high *DLL4* expression in a subset of these specimens. Moreover, in a small collection of patient-derived T-ALL xenografts (PDTALL), we identified one in which DLL4 was expressed at the cell membrane. In this PDTALL, exposure to demcizumab, a blocking antibody against human DLL4 tested in clinical trials in patients with solid cancer 8, 9, had similar effects as global Notch pathway inhibition using the potent γ-secretase inhibitor dibenzazepine (DBZ). This demonstrated that in this PDTALL model, Notch pathway activity depends on DLL4 expression on T-ALL cells.

In summary, we demonstrated that spleen is crucial for DLL4-driven T-ALL generation in the Tg8 mouse model, and that DLL4 expression on T-ALL cells promotes Notch activity in human T-ALL, validating our preclinical findings.

## Results

### In Tg8 mice, circulating CD4^+^CD8^+^ cells are not exported from the thymus, but from the spleen

Previous studies showed that in different mouse models of DLL4-driven T-ALL, non-tumoral circulating CD4^+^CD8^+^ cells appear before disease onset 6, 7. However, the source of these CD4^+^CD8^+^ cells and their role in T-ALL are unknown. Therefore, we characterized their appearance in Tg8 mice.

**Figure 1:**
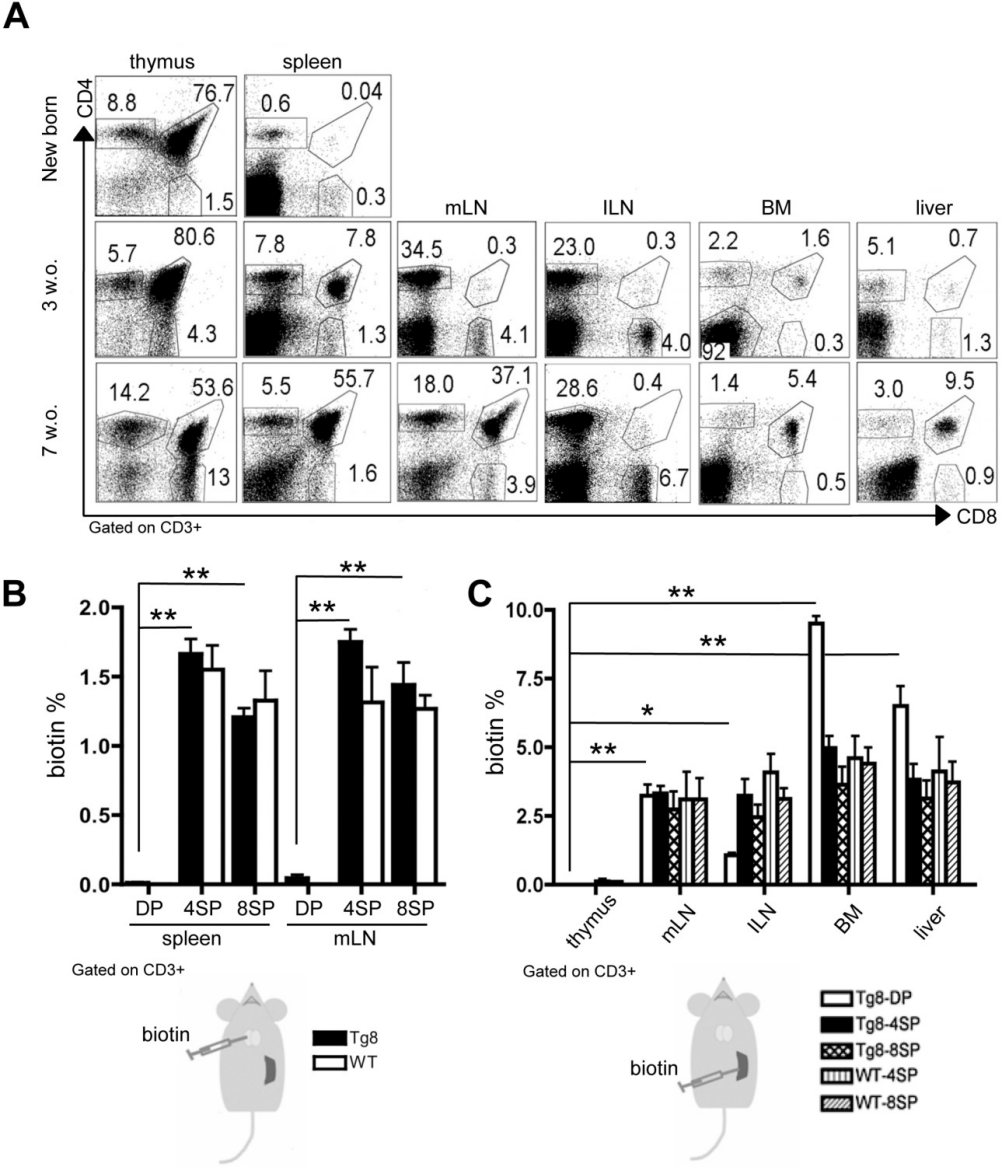
** Circulating CD4^+^CD8^+^ cells in Tg8 mice are not exported from the thymus but from the spleen. A)** Kinetics of CD4^+^CD8^+^ cell appearance in thymus, spleen, mesenteric lymph nodes (mLN), inguinal lymph nodes (ILN), bone marrow (BM), and liver of Tg8 mice determined by flow cytometry (data are representative of n = 4 mice for each indicated time point). Cells were positively gated using CD3 and analyzed for the expression of CD4 and CD8. **B)** Biotin was injected into the thymus of 5-week-old Tg8 or wild type (WT) mice. 24 h later, biotin^+^ cells recently emigrated from the thymus were identified in spleen and mLN by staining with an anti-biotin antibody. Data are the mean ± SEM (n = 4 mice per genotype). **C)** Biotin was injected in the spleen of 5-week-old Tg8 or WT mice. 24 h later, biotin^+^ cells recently emigrated from the spleen were identified in thymus, mesenteric lymph nodes (mLN), inguinal lymph nodes (ILN), bone marrow (BM), and liver. Data are the mean ± SEM (n = 3 mice per genotype). In (B) and (C) cells were gated using CD3 and analyzed for streptavidin and CD4 and CD8 expression. In (B) and (C): * *p* < 0.05 ** *p* < 0.01 (Mann-Whitney test). DP, CD4^+^CD8^+^ double-positive cells; 4SP, CD4^+^ single-positive cells; 8SP, CD8^+^ single-positive cells; w.o., week-old.

In newborn Tg8 mice, we did not detect any CD4^+^CD8^+^ cell outside the thymus (Figure 1A). Conversely, in 3-week-old mice, we observed CD4^+^CD8^+^ cells in the spleen, and to a lower extent also in other organs: mesenteric and inguinal lymph nodes (mLN and ILN), bone marrow (BM), and liver. In 7-week-old mice, CD4^+^CD8^+^ cells were the most abundant lymphoid cell population in spleen, suggesting that spleen is the main organ in which such cells accumulate outside the thymus in Tg8 mice.

Next, to determine whether these peripheral CD4^+^CD8^+^ cells originated in the thymus, we injected biotin in the thymus of 5-week-old wild type (WT) and Tg8 mice. Biotin uniformly labeled thymocyte populations (CD4^+^CD8^+^, and CD4^+^ or CD8^+^ single-positive cells) (Figure S1A). At 24 h post-injection, we observed biotin^+^/CD4^+^ and biotin^+^/CD8^+^ cells in spleen and mLN in both genotypes. Conversely, double-positive CD4^+^CD8^+^ cells in spleen and mLN were not labeled by biotin (Figure 1B-S1B), suggesting that they were not exported from the thymus or were exported much more slowly than mature T cells.

As spleen was the first peripheral organ showing CD4^+^CD8^+^ cells, we hypothesized that they could be generated in this organ. Therefore, we injected biotin in the spleen of WT and Tg8 mice, and 24h later we tracked biotin^+^ cells. We found biotin^+^/CD4^+^CD8^+^ cells in most of the organs examined, except thymus (Figure 1C), indicating that, unlike thymic CD4^+^CD8^+^ cells, splenic CD4^+^CD8^+^ cells can rapidly circulate to other peripheral organs. As most splenic CD4^+^ and CD8^+^ single-positive cells belonged to the recirculating pool of mature T cells, we found biotin^+^/CD4^+^ and biotin^+^/CD8^+^ single-positive cells in both WT and Tg8 peripheral organs after intrasplenic biotin injection (Figure 1C-S1C).

To confirm these results, we transferred a mixture of CD4^+^CD8^+^ double-positive and CD4^+^ single-positive T cells from donor Tg8 spleens to the spleen or thymus of recipient Tg8 mice. We could distinguish the three cell types (donor CD4^+^CD8^+^, donor single-positive, and recipient T cells) using Thy1 allelic markers (Figure S2A). Analysis of the distribution of the injected cells after 3 days showed that both CD4^+^CD8^+^ and CD4^+^ cells injected in the spleen migrated to mLN (Figure S2B red square and S2C). Conversely, only CD4^+^ single-positive cells, but not CD4^+^CD8^+^ cells injected in the thymus migrated to mLN (Figure S2B, blue square and S2C). Analysis in CD4^+^CD8^+^ cells (from the spleen or thymus), as well as in CD4^+^ single-positive cells, showed that both splenic and thymic CD4^+^CD8^+^ cells weakly expressed CCR7 and CD62L compared with mature CD4^+^ cells (Figure S2D). These markers are needed in mature single-positive T cells to egress the thymus and migrate into LN 10, 11.

Altogether, these findings indicated that in Tg8 mice, spleen accumulates and exports CD4^+^CD8^+^ cells.

### All early T-cell developmental stages are found in the spleen

**Figure 2:**
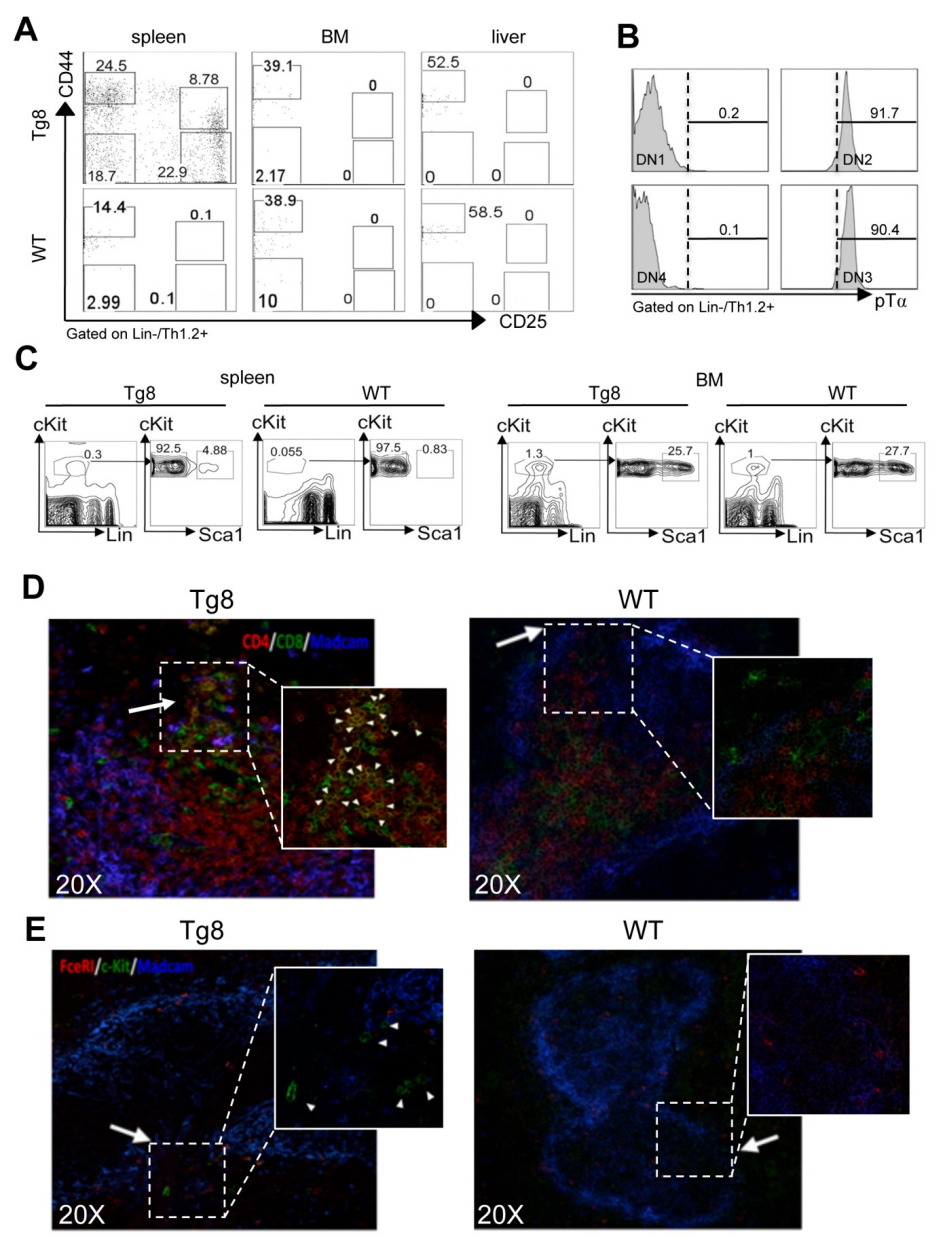
** All early T-cell developmental stages are found in the spleen of Tg8 mice. A)** Spleen, bone marrow (BM) and liver tissues from 7-week-old Tg8 animals were homogenized and stained. DN cells were negatively gated on lineage (Lin = CD3/4/8/19/11c/F4/80/Gr1/FcεRI/TCRγδ), positively gated on Thy1.2^+^, and classified as DN1-DN4 with anti-CD25 and -CD44 antibodies. Data are representative of n = 4 mice for each genotype. **B)** DN1/DN2/DN3/DN4 cells from spleen of Tg8 mice identified as in (A) were stained with antibodies against pre-TCRα (pTα). Representative data from n = 4 mice. **C)** Spleen and bone marrow cells from 3-week-old Tg8 and WT animals were homogenized and stained. LSK cells were negatively gated on lineage (Lin = CD3/4/8/19/11c/F4/80/Gr1/FcεRI/TCRγδ), positively gated on cKit expression, and then analyzed for Sca1 expression. Data are representative of n=4 mice for each genotype. CD4^+^CD8^+^ cells **D)** and progenitor LSK cells **E)** are found at the bridging channels (BC) of the marginal sinus endothelia surrounding the peri-arteriolar lymphoid sheaths. Frozen spleen sections from 14-day-old Tg8 and WT mice were fixed and stained with anti-CD4, anti-CD8, anti-MADCAM, anti-c-Kit, anti-FcεRI and secondary antibodies conjugated with fluorescent dyes. Arrows indicate BCs, which are the discontinuities of the marginal sinus endothelia stained with MADCAM in both (D) and (E). Arrowheads indicate CD4^+^CD8^+^ (D) and LSK cells (E). Data are representative of n = 5 mice per genotype.

The findings on peripheral CD4^+^CD8^+^ cell accumulation and export kinetics suggested that spleen could be a preferential site of extrathymic CD4^+^CD8^+^ T-cell generation in Tg8 mice. Therefore, we investigated the presence of double-negative cells (i.e. CD4^+^CD8^+^ progenitors) during T-cell development. Analysis of CD44 and CD25 expression in the CD3/4/8/19/11c/F4/80/Gr1/FcεRI/TCRγδ (LIN)-negative/Thy1-positive population indicated that all double-negative precursors (DN1 to 4) were present in spleens of Tg8 mice, but not of WT animals (Figure 2A). We did not detect any double-negative cell in BM or liver (Figure 2A), indicating that spleen might favor DLL4-driven T-cell development *in vivo* outside the thymus in Tg8 mice. Moreover, in Tg8 mice, DN2 and DN3 spleen cells expressed pre-TCRα, confirming their identity as double-negative T cells (Figure 2B).

Next, we wanted to identify the multipotent hematopoietic progenitors that could give rise to double-negative cells in the spleen of Tg8 mice. Flow cytometry analysis showed that in 3-week-old Tg8 mice, spleen already contained a population of cells with multipotent progenitor markers (LSK cells: LIN^-^, Sca1^+^, cKit (i.e., CD117)^+^ cells (Figure 2C). The number of LSK cells was 5 times lower in spleens from WT animals than from Tg8 mice (Figure 2C), indicating that LSK cells accumulated better in Tg8 spleens. As expected, the percentage of LSK cells was higher in BM than in spleen; however, unlike spleen, the proportion of LSK cells in BM was similar in Tg8 and WT mice (Figure 2C).

We next assessed whether in Tg8 mice, spleen could support development of WT T cells. To this aim, we injected WT BM cells in the spleen of Tg8 and WT mice. At day 10 post-injection, we detected donor WT CD4^+^CD8^+^ cells only in the spleen of Tg8 mice, but not of WT animals (Figure S2E).

All these results are in accordance with our seminal study showing that transfer of Tg8, but not WT single-positive CD4^+^ cells can promote CD4^+^CD8^+^ cell development in nude mice 6.

### LSK cells accumulate in the bridging channels of splenic white pulp

To better characterize the early events of CD4^+^CD8^+^ cell generation in spleen, we analyzed by immunofluorescence the spleen of 14-day-old Tg8 and WT mice. In Tg8 spleens, CD4^+^CD8^+^ cell accumulation started immediately adjacent to bridging channels (BC), which are identified by the presence of marginal sinus-lining MADCAM1^+^ endothelial cells (Figure 2D). This is exactly the site where circulating T cells enter the spleen white pulp 12. LSK cells (identified as cKit^+^ FcεRI^-^) also were near the BC in Tg8 spleens (Figure 2E). This suggests that in Tg8 spleens, BCs might provide the proper environment for the contact between LSK cells and DLL4^+^ positive T cells to initiate their differentiation along the T-cell lineage. In Tg8 spleens, we did not detect any LSK cell outside the BC area, including the red pulp. Moreover, we did not detect LSK cells in WT spleens by immunofluorescence (Figure 2E). Together with the flow cytometry analysis (Figure 2C), this indicated that the WT spleen environment does not favor the accumulation of circulating LSK cells. CD4^+^CD8^+^ cell number strongly increased in 35-day-old Tg8 mice (Figure S3A).

One simple explanation for T-cell development in spleen could be its very high density of T cells (hence, more DLL4 ligands) compared with other lymphoid organs. As T-cell density in LN is at least as high as in spleen, we compared the location/density of CD4^+^CD8^+^cells in mLN and spleen in 7-week-old mice, because at this age mLN was the second organ after spleen to harbor CD4^+^CD8^+^ cells (Figure 1A). The density of single-positive cells (hence, DLL4 density) in mLN was at least as high (if not more) as in spleen (compare Figure S3A and Figure S3B), ruling out the hypothesis of higher T-cell density in spleen as a factor favoring CD4^+^CD8^+^ cell differentiation in this organ. However, in mLN, CD4^+^CD8^+^ cells were almost completely segregated from CD4^+^ and CD8^+^ single-positive cells in Tg8 mice. Specifically, CD4^+^CD8^+^ cells were clustered in the medullary sinuses, whereas single-positive cells occupied the regular T-cell areas in the subventricular zones (Figure S3B). This finding and the low CD62L and CCR7 expression on peripheral CD4^+^CD8^+^ cells (Figure S2D) strongly suggest that unlike normal T cells, CD4^+^CD8^+^ cells entered the mLN through the afferent lymph rather than through high endothelial venules, leading to the spatial segregation of double- and single-positive cells. This also confirmed that in Tg8 mice, peripheral CD4^+^CD8^+^ cells are not generated in mLN.

### Lack of positive selection of CD4^+^CD8^+^ cells generated in spleen

**Figure 3:**
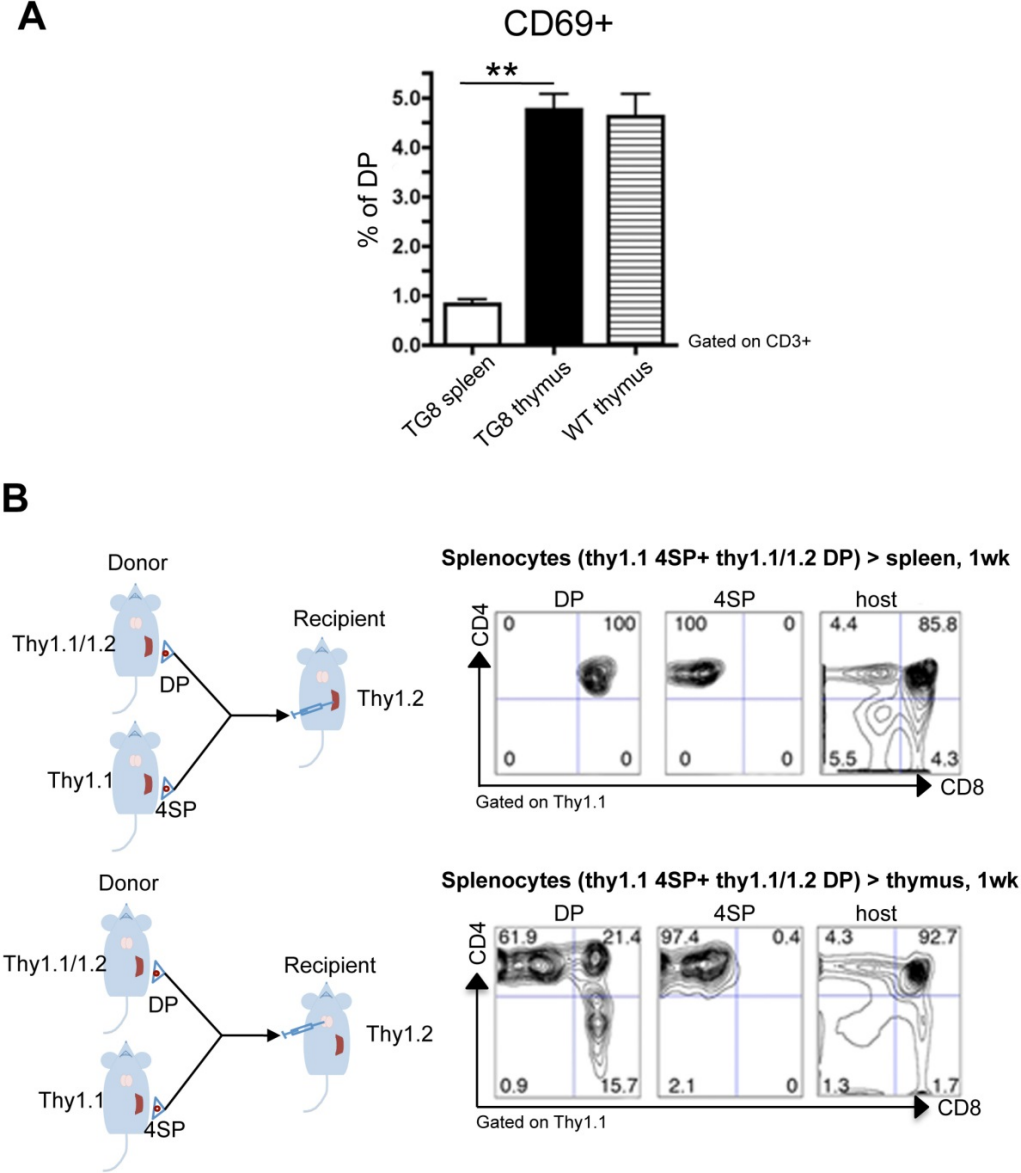
** Lack of positive selection of CD4^+^CD8^+^ splenic Tg8 cells. A)** Spleen, and thymus tissues from 7-week-old Tg8 animals were homogenized and stained. Cells were gated for CD3 expression, and CD3^+^ cells analyzed for CD4, CD8 and CD69 expression. The y-axis shows the percentage of CD69^+^ cells among all double-positive (DP) CD4^+^CD8^+^ cells. Data are the mean ± SEM (n = 5 mice per genotype). **B)** Splenic CD4 single-positive (4SP) cells from Tg8 Thy1.1 animals and CD4^+^CD8^+^ (DP) cells from 7-week-old Tg8 Thy1.1/1.2 animals were sorted separately and co-injected in spleen (top panels) or thymus (bottom panels) of Tg8 Thy1.2 recipient animals. One week later, cells were collected from the spleen or thymus of recipient mice, and stained with anti-CD4, -CD8, -Thy1.1 and -Thy1.2 antibodies, cells were positively gated in Thy1.1 for donor cells or gated Thy1.2 host cells, and analyzed for CD4 and CD8. Data are representative of n = 5 mice per group. ** *p* < 0.01 (Mann-Whitney test).

We hypothesized that CD4^+^CD8^+^ cell accumulation in spleen was due to a defect in the positive selection of new CD4^+^CD8^+^ cells generated in this organ. One of the early events in the positive selection of T cells is CD69 upregulation 13-15. Indeed, around 5% of WT and Tg8 thymic CD4^+^CD8^+^ cells expressed CD69 (Figure 3A). This CD69^+^/CD4^+^CD8^+^ cell population was strongly reduced in Tg8 spleens (Figure 3A), suggesting that spleen cannot positively select CD4 or CD8 single-positive cells or that splenic CD4^+^CD8^+^ cells have intrinsic defects that arrest them at this stage. To rule out this second hypothesis, we performed transfer experiments in which we injected allelically marked splenic double- and single-positive cells from Tg8 mice in the spleen or thymus of recipient Tg8 mice. As expected, CD4^+^ single-positive cells were stable and remained single-positive after injection in thymus or spleen (Figure 3B “4SP”). Consistent with all our previous data, donor CD4^+^CD8^+^ cells became single-positive cells only after injection in thymus (Figure 3B lower panel “DP”), but not after injection in spleen (Figure 3B top panel “DP”).

Altogether, our data demonstrate that in Tg8 mice, extrathymic CD4^+^CD8^+^ cells generated in spleen do not have intrinsic developmental deficiency; however, they are not in the right environment, therefore they cannot be positively selected, and accumulate in the periphery. Hence, we hypothesized that splenic CD4^+^CD8^+^ cells could be at the origin of T-ALL in our model of DLL4-driven T-ALL.

### Spleen is required for extrathymic CD4^+^CD8^+^ cell generation and T-ALL development in Tg8 mice

**Figure 4:**
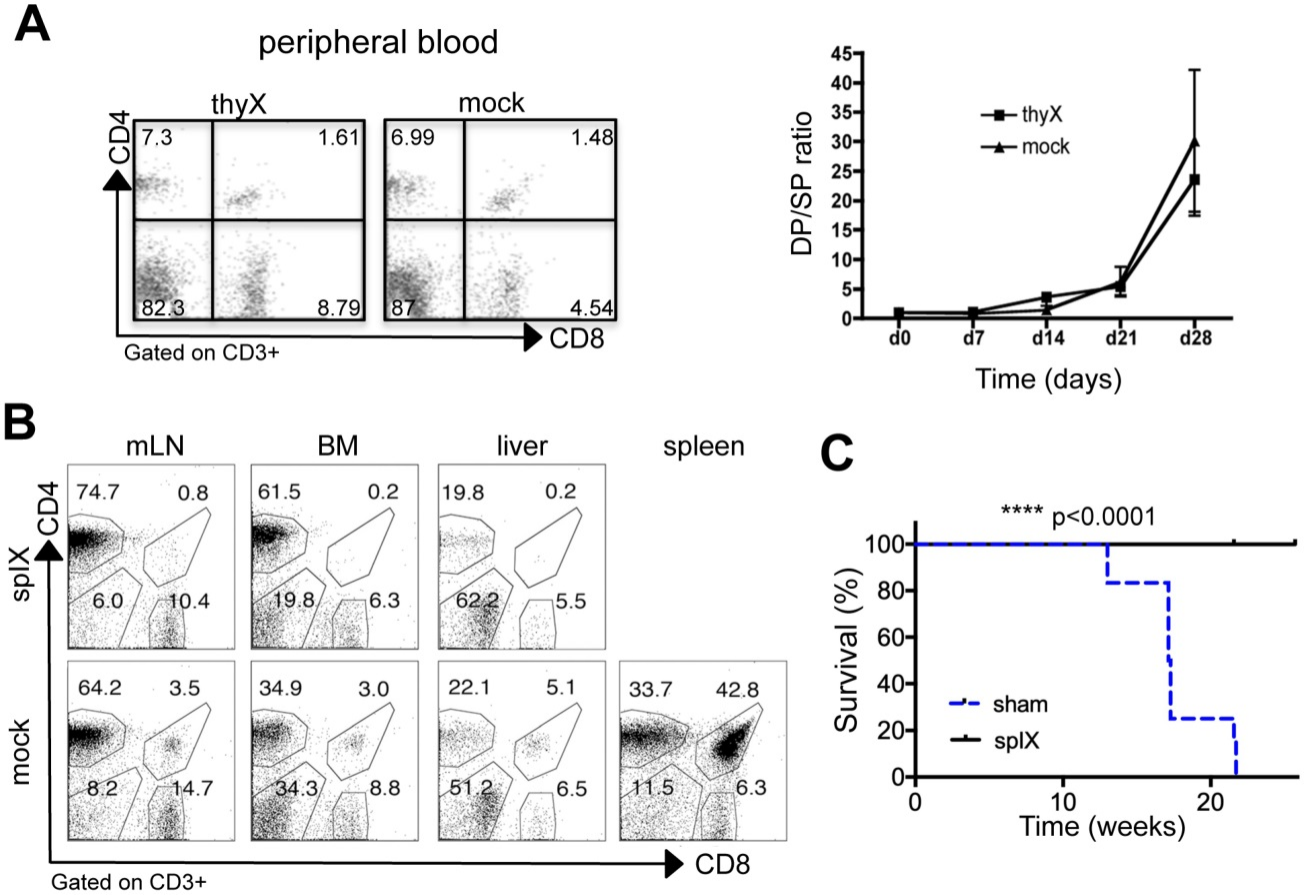
** Spleen is required for extrathymic CD4^+^CD8^+^ cell development in DLL4-driven T-ALL. A)** Three-week-old Tg8 mice underwent thymectomy (thyX) or sham surgery (mock) and blood cells were collected and stained for CD3, CD4 and CD8 every week after surgery. Cells were gated on CD3 expression, and analyzed for CD4 and CD8 expression. The left panel shows a representative analysis at week 3 after surgery. The right panel shows the CD4^+^CD8^+^/CD4^+^ (DP/4SP) ratio increase after surgery (n = 6 mice per treatment). **B)** Three-week-old pre-tumoral Tg8 mice underwent splenectomy (splX) or sham surgery (mock). One month after surgery, mice were sacrificed and CD4^+^CD8^+^ cell accumulation in mesenteric lymph nodes (mLN), bone marrow (BM), liver, and spleen of Tg8 mice determined by flow cytometry (data are representative of n=4 mice for each indicated time point). Cells were positively gated using CD3 and analyzed for the expression of CD4 and CD8. Data are representative of n = 4 mice per treatment. **C)** Survival curves of mice that underwent splenectomy (splX) or sham surgery (sham) at 3 weeks of age. n = 12 mice per treatment coming from 2 different experiments with 5 and 7 mice per group, respectively. In the first experiment, splenectomized mice were killed when the last sham-operated mouse died of T-ALL and they appear as censored (with a tick mark). In the second experiment, splenectomized mice were kept alive for another month after the last sham-operated mouse died, and also appear as censored (with a tick mark). Kaplan-Meier survival curves were analyzed with the Gehan-Breslow-Wilcoxon test (*p* = 0.000012). **** *p* < 0.0001.

First, we wanted to prove that thymus was not required for DP cell accumulation at the periphery in Tg8 mice. To this aim, we performed thymectomies in 3-week-old Tg8 mice. In accordance with all data presented above, we observed that the fraction of extrathymic CD4^+^CD8^+^ cells was not affected by thymus removal (compared with sham surgery, Figure 4A). This formally demonstrates that in Tg8 mice, peripheral CD4^+^CD8^+^ cells are not generated in the thymus. All Tg8 mice (surgery and sham surgery) developed T-ALL with identical timing (data not shown), indicating that extrathymic CD4^+^CD8^+^ cells might be needed for T-ALL development.

Then, to determine the role of spleen in Tg8 mice, we splenectomized 3-week-old Tg8 mice. Circulating CD4^+^CD8^+^ cells were below detection level in splenectomized mice, while they accumulated normally in sham-operated Tg8 mice (Figure 4B). Neither CD4^+^CD8^+^ cell development in thymus (Figure S4A) nor DLL4 expression in peripheral Tg8 T cells was affected by splenectomy (Figure S4B). Therefore, we concluded that the spleen is required for extrathymic development in the Tg8 mouse model of DLL4-driven T-ALL. Strikingly, even one month after all sham-operated mice had died due to T-ALL, splenectomized Tg8 mice were fully protected against leukemia development (Figure 4C). These data indicate that extrathymic CD4^+^CD8^+^ cells are at the origin of T-ALL in Tg8 mice.

To demonstrate that spleen is specifically required for DLL4-driven T-ALL development, we used a Notch Intracellular Domain (NICD)-driven T-ALL mouse model (i.e. EF1a-N1IC R26-Cre ER mice) in which *Notch1* NICD is preceded by Flox/STOP/Flox 16. We transplanted BM from EF1a-N1IC R26-Cre ER mice in lethally irradiated mice, and after 4 weeks, we splenectomized or sham-operated these mice. Upon induction of NICD expression by treatment with tamoxifen at week 2 post-surgery, all splenectomized and sham-operated mice succumbed to T-ALL at the same time (data not shown), indicating that spleen is not required for T-ALL development when Notch can be activated in the absence of DLL4. At early stages (i.e. at week 2 after NICD induction), we started to detect CD4^+^CD8^+^ cells in peripheral blood from sham-operated mice and also, but to a lower extent, in splenectomized mice (Figure S4C). This suggested that spleen is a preferential location for extrathymic CD4^+^CD8^+^ cell development even upon NICD expression.

Altogether, our results demonstrate that splenic extrathymic T-cell development is at the origin of T-ALL in the DLL4-driven T-ALL Tg8 mouse model.

### In DLL4-expressing T-ALL, demcizumab has the same therapeutic effect as γ-secretase inhibition

**Figure 5:**
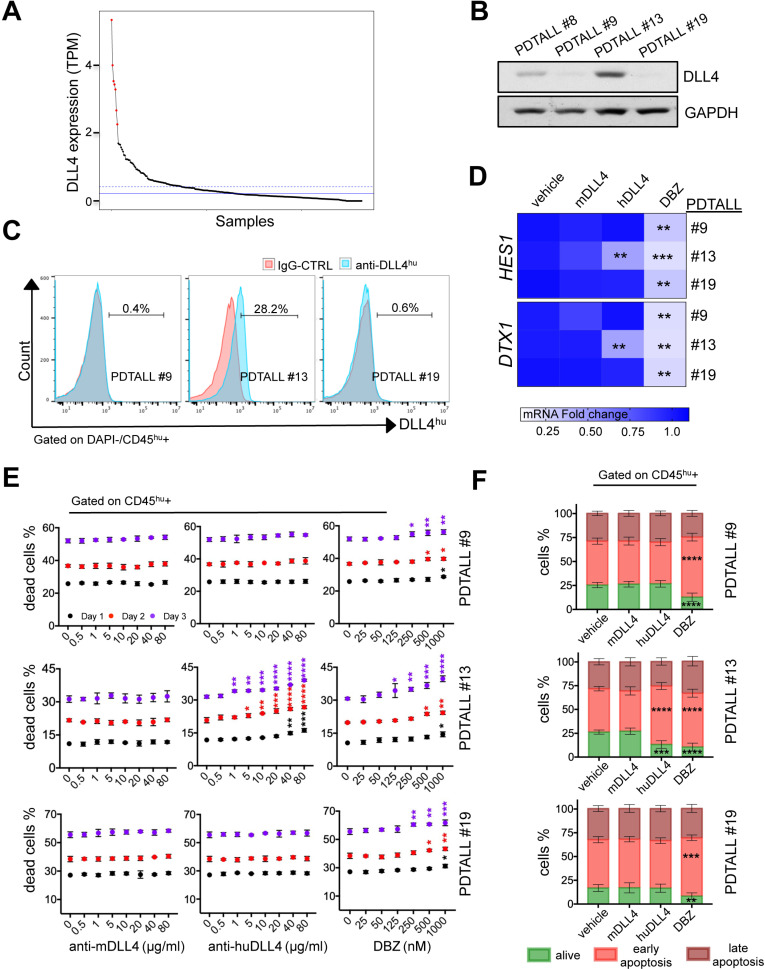
** In DLL4-expressing T-ALL, demcizumab treatment showed the same therapeutic effect as γ-secretase inhibition. A)** Normalized TPM expression levels of DLL4 in 264 T-ALL patient samples (Mullighan dataset) in decreasing order. Samples with the highest expression level (z score > 2) are highlighted in red (top 2.6%). The dashed blue horizontal line indicates the mean expression level, and the solid blue horizontal line indicates the median expression level across all samples. **B)** Analysis of DLL4 expression by western blotting in four different patient-derived T-ALL (PDTALL). **C)** The spleens of moribund NRG mice harboring different PDTALL cells were homogenized and stained. Cells were gated negatively (DAPI) and positively for CD45 expression, and analyzed for the expression of human DLL4 or the isotype IgG control. PDTALLs were expanded in mice before performing the *in vitro* assays presented in (D), (E) and (F). **D)** Analysis of *HES1* and *DTX1* mRNA expression in the indicated PDTALL cells incubated with vehicle (control), 20 µg/ml of murine anti-DLL4 antibody, 20 µg/ml of human anti-DLL4 antibody (demcizumab), or 500 nM of DBZ for 48 h. Data (treatments vs control) were compared by one-way ANOVA for each PDTALL, n = 3 each condition. **E)** Viability of PDTALL9 (upper), 13 (middle) and 19 (low) cells incubated with the indicated doses of murine anti-DLL4 antibody, human anti-DLL4 antibody (demcizumab), or DBZ for 1, 2 and 3 days. Cells were gated using a human anti-CD45 antibody, and cell death analyzed by DAPI staining. Data (treatments vs control) were compared by two-way ANOVA for each day; n = 2 per condition. **F)** Apoptosis analyses of PDTALL9 (upper), 13 (middle) and 19 cells (lower) performed by flow cytometry after staining with AnnexinV and DAPI following incubation with vehicle (control), murine anti-DLL4 antibody, human anti-DLL4 antibody, or DBZ as in (D). Data (treatments vs control) were compared by two-way ANOVA, n = 3 per condition. * *p* < 0.05, ** *p* < 0.01, *** *p* < 0.001, **** *p* < 0.0001.

To assess the clinical significance of our findings in Tg8 mice, we first analyzed *DLL4* mRNA expression in a publicly available database of human T-ALL specimens 17 after transforming the TPM values for *DLL4* expression into Z-scores. We found that 2.6% of these samples had a Z-score > 2 (in red in Figure 5A). Pairwise Pearson correlation analysis of TPM values did not highlight any significant correlation between *DLL4* expression and other members of the Notch signaling pathway (*NOTCH1*, *NOTCH3*, *HES1*, *DTX1* and *MYC*) (Figure S5). Moreover, unsupervised hierarchical clustering of the samples from this dataset revealed that four of the seven samples with the highest DLL4 expression (Z-score > 2) clustered together (Figure S6). Finally, a completely unsupervised approach to identify mutations enriched in those seven T-ALL samples with the highest *DLL4* mRNA expression did not identify any significant association with any mutation. Still, in five of the seven T-ALL samples mentioned above, we found *NOTCH1* somatic mutations, but the available data did not allow us to determine the heterozygosity frequency in this subset compared with the DLL4-negative samples.

In summary, we conclude that a small subset of T-ALL samples displays high *DLL4* expression levels compared with the other specimens of this dataset 17. However, we could not determine whether in these samples DLL4 was expressed at the cell membrane of malignant cells or in other cell types (e.g. stromal cells).

Therefore, to evaluate whether in human T-ALL, DLL4 is expressed at the cell membrane, we analyzed DLL4 protein expression in a small group of already available patient-derived T-ALL (PDTALL) models: PDTALL 8, 9, 13 and 19. Some of these PDTALLs harbor mutations in *NOTCH1* (8 and 19) and *FBXW7* (19), whereas the others carry WT *NOTCH1* (9 and 13) and* FBXW7* (8, 9 and 13) 18. We injected PDTALL cells in immunodeficient NRG mice and after 3 weeks, we isolated cells from enlarged spleens and analyzed DLL4 expression by western blotting. DLL4 expression was high only in PDTALL13 (Figure 5B). Then, we analyzed by flow cytometry DLL4 expression in PDTALL13 cells and in PDTALL9 and 19 cells, as negative controls. We found that DLL4 was expressed at the membrane in 28% of PDTALL13 cells (gated with an anti-human CD45 antibody, Figure 5C). Conversely, we did not detect any DLL4 expression in PDTALL9 and 19 cells (Figure 5C).

It was previously shown that inhibition of murine DLL4 expressed by stroma cells hampers the Notch pathway and the viability of some PDTALL models *in vivo* 4. However, to the best of our knowledge, this is the first time that DLL4 expression was detected in T-ALL cells. Therefore, we wanted to determine whether human DLL4 is required for Notch pathway activity and survival of PDTALL13 cells. To this aim, we incubated PDTALL13 cells *in vitro* with vehicle, demcizumab (a blocking anti-human DLL4 antibody), a murine anti-DLL4 antibody (negative control), or the potent γ-secretase inhibitor DBZ, a pan-Notch inhibitor (positive control). We used PDTALL9 and 19 cells as negative controls for demcizumab treatment. The expression levels of *DTX1* and *HES1* were not significantly different in cells incubated with the mouse anti-DLL4 antibody or vehicle in all tested PDTALL models. Conversely, DBZ led to a reduction of *HES1* and *DTX1* expression levels in all tested PDTALL models. Moreover, demcizumab efficiently decreased *HES1* and *DTX1* levels in PDTALL13 cells, but showed no effect in PDTALL9 and 19 cells (Figure 5D). This confirmed that the DLL4 detected at the membrane of PDTALL13 cells is of human origin, and indicated that the Notch pathway is activated by DLL4 in PDTALL13 cells. Analysis of cell survival showed that the murine anti-DLL4 antibody did not cause death of human CD45^+^ cells in the three PDTALL models, not even at the highest concentration (80 µg/ml) (Figure 5E). Conversely, DBZ (positive control for Notch inhibition) promoted cell death in all three PDTALL models, but with different kinetics. Demcizumab promoted cell death only in PDTALL13 cells, and very efficiently. Indeed, we observed a cell death increase (compared to vehicle) with only 1 µg/ml. All these data confirmed the specificity of demcizumab towards T-ALL cells that overexpress DLL4 (Figure 5C). Moreover, as the Notch pathway provides pro-survival signals to T-ALL cells grown *in vitro* 4, 19, our cell death data further suggested that inhibiting DLL4 signaling in PDTALL13 cells abolishes Notch signaling. Finally, we also observed that the murine anti-DLL4 antibody caused cell death in murine cells (i.e. cells negative for human CD45), but only at high concentration (40-80 µg/ml). Conversely, demcizumab (negative control) did not promote cell death in murine cells, and DBZ (positive control) showed a similar murine cell death profile in all three PDTALL models (Figure S7).

To better investigate cell death in our experimental setting, we quantified Annexin V/DAPI staining by flow cytometry in the three PDTALL models after incubation with DBZ or antibodies against murine and human DLL4. Incubation with DBZ increased the percentage of Annexin V-positive cells (i.e. early apoptosis) in the three PDTALL models (Figure 5F), confirming the known role of the Notch pathway in T-ALL survival 4, 19. Moreover, in PDTALL13 cells, the percentage of cells in early apoptosis was similarly increased after incubation with demcizumab. This confirmed that the Notch pathway is dependent on DLL4 signaling in this model (Figure 5F and Figure S8).

**Figure 6:**
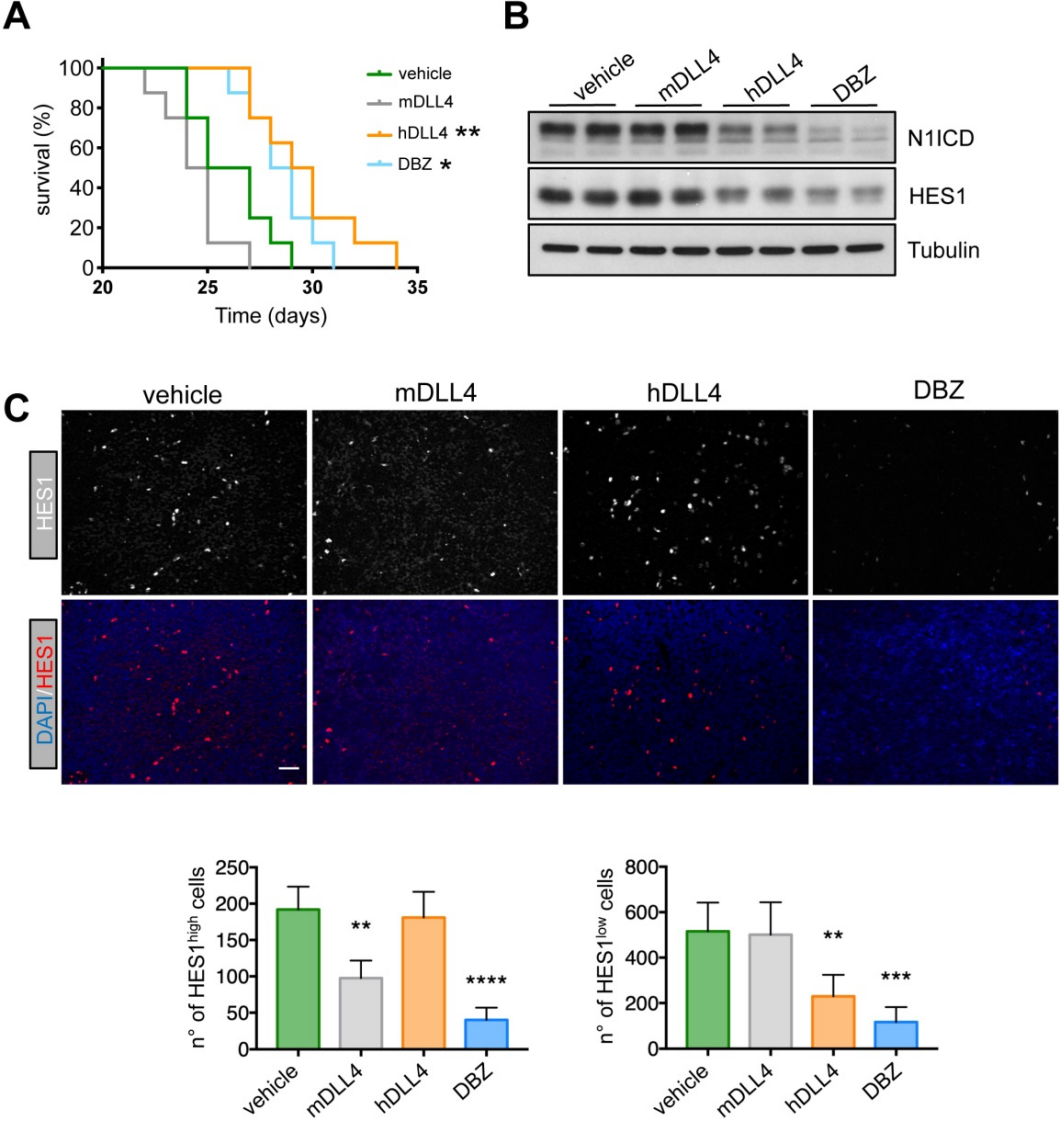
** Therapeutic effect of demcizumab in mice harboring DLL4-expressing T-ALL cells. A)** PDTALL13 cells were injected IV in NRG mice that were then treated with vehicle (IgG, n = 8), DBZ (n = 8), murine anti-DLL4 (n = 8), or human anti-DLL4 antibodies (demcizumab, n = 8). The Y-axis shows the percentage of alive animals and the X-axis the days after treatment. Data were compared with the Gehan-Breslow-Wilcoxon test: vehicle vs DBZ (p = 0.02), vehicle vs demcizumab (p = 0.006), murine anti-DLL4 antibody vs DBZ (p = 0.0003), and murine anti-DLL4 antibody vs demcizumab (p = 0. 0002). The other comparisons were not significant. Asterisks in the figure are for the comparison with the vehicle group. **B)** Protein expression of the indicated proteins by western blotting of whole cell extracts from spleens of NRG mice treated as in (A). **C)** Formalin fixed-paraffin embedded spleen sections from NRG mice treated as in (A) were stained with anti-HES1 antibodies, DAPI and secondary antibodies conjugated with fluorescent dyes. Four random photographs at 20X magnification were taken for each condition and the number of HES1^high^ cells (left) and HES1^low^ cells (right) is presented below. Statistical analyses (treatment vs control) were performed by one-way ANOVA for each PDTALL line. * *p* < 0.05, ** *p* < 0.01, *** *p* < 0.001, **** *p* < 0.0001.

To determine whether these findings could be translated to the clinic, we injected PDTALL13 cells in irradiated NRG mice and, 24 h later, we randomized mice in four treatment groups: control (vehicle), DBZ, murine anti-DLL4 antibody, and demcizumab. In accordance with our *in vitro* data, the median survival of mice treated with vehicle (control) or the mouse anti-DLL4-antibody was not significantly different (26 and 24.5 days, respectively) (Figure 6A). Conversely, survival was similarly increased in mice treated with DBZ or demcizumab (28.5 and 29.5 days respectively) compared with the two previous groups (Figure 6A). To test whether the different treatments reached their targets, we analyzed by western blotting spleen samples from mice harboring PDTALL13 cells and treated with vehicle, murine or human anti-DLL4, or DBZ. The expression levels of Notch intracytoplasmic domain (N1ICD) and HES1 were decreased in samples from mice treated with demcizumab and DBZ compared with mice treated with the murine anti-DLL4 antibody or vehicle (Figure 6B). These data further demonstrated that DLL4 signaling is the main source of Notch activity in PDTALL13. Finally, and to demonstrate that the murine anti-DLL4 antibody also reached its target, we analyzed HES1 expression by immunofluorescence in spleen cells after treatments. First, we observed two cell types: one with high HES1 expression (few cells), and one with low HES1 expression (many cells) (Figure 6C). DBZ treatment decreased both HES1 expression patterns. Conversely, the mouse anti-DLL4 antibody decreased only the number of HES1^high^ cells, whereas demcizumab treatment decreased only the number of HES1^low^ cells. These findings strongly suggested that HES1^high^ cells are murine and HES1^low^ cells are human T-ALL cells. To prove this, we co-stained cells with an antibody against human vimentin, an antibody against human and murine vimentin, an anti-HES1 antibody, and DAPI. HES1^high^ cells were only stained with the vimentin antibody against murine and human cells (Figure S9). Conversely, the abundant HES1^low^ cells were stained by both anti-vimentin antibodies. This confirmed that HES1^high^ cells are murine and HES1^low^ cells are human, even more, also that murine anti-DLL4 antibody worked in our experimental setting.

Taken together, our data show that DLL4 is strongly expressed in a small subset of T-ALL and that inhibition of human DLL4 signaling in a DLL4-positive PDTALL inhibits the Notch pathway and reduces T-ALL cell survival, as observed upon γ-secretase inhibition. All the human data validated our findings in our preclinical model of DLL4-driven T-ALL and broaden our knowledge on this disease.

## Discussion

In this work we demonstrated that spleen is crucial in DLL4-driven T-ALL. We think that this might be explained by a combination of factors, particularly the fact that HSCs and T cells are closer in spleen than in other peripheral lymphoid organs, and the presence of a specific microenvironment (cytokines, cell-cell interactions) in spleen. A previous study in which murine NICD-driven T-ALL cells were injected in nude mice showed that the spleen has a role in T-ALL cell colonization. Indeed, splenectomized mice survived longer than sham-operated control mice, although all mice ultimately died 20. Here, we found that spleen does not play a role, or only a limited one, in NICD-driven T-ALL because splenectomy did not protect against T-ALL development. Conversely, DLL4-driven T-ALL development was inhibited by splenectomy, demonstrating differences between these models of Notch-induced T-ALL. Currently, we cannot rule out that besides the extrathymic T-cell development we reported here, in Tg8 mice spleen could provide the perfect environment for T-ALL precursor survival. Our data also highlight the importance of the tight temporal and spatial regulation needed for T-cell development because the lack of DLL4-driven splenic cell positive selection allowed them to survive. Moreover, as these cells express DLL4 at the membrane, they also generate more CD4^+^CD8^+^ cells that accumulate until the appearance of additional mutations required for T-ALL 6. The crucial role of spleen in our DLL4-driven T-ALL model was strengthened by the finding that mice in which spleen was surgically removed were in good health even one month after the last sham-operated mouse died due to T-ALL. However, we cannot rule out that at later stages, they could develop the disease.

The percentage of T-ALL patients with elevated Notch pathway activity is higher than that of patients with somatic mutations in *NOTCH1* and/or *FBWX7,* suggesting that other mechanisms might support NOTCH hyperactivation in T-ALL 21, 22. For instance, increased NOTCH1 levels induce T-ALL in *Ccnc^-/-^* mice 23, and ectopic DLL4 expression promotes T-ALL in mice 6, 7. Although *CCNC* loss of function mutations have been reported in some patients with T-ALL 23, the involvement of membrane-bound DLL4 in cancer cells remained elusive until our present proof of concept study showing DLL4 involvement in the human disease. Interestingly, DLL4-expressing PDTALL13 cells do not harbor any *NOTCH1* or *FBWX7* mutation 18. However, we could not find any correlation between T-ALL samples with higher DLL4 expression and *NOTCH1* or *FBWX7* mutational status in the T-ALL specimens analyzed in Figure 5A 17. Therefore, more work is needed to clarify the relationship between DLL4 expression and Notch pathway mutations in T-ALL. Finally, our work fits with a previous study showing that JAGGED1, another Notch ligand, also plays a role in T-ALL 24.

Previous work demonstrated that in nude mice harboring PDTALL cells, murine anti-DLL4 antibodies hamper T-ALL growth 4; however, to the best of our knowledge, this is the first study showing that human T-ALL cells can express DLL4 to activate the Notch pathway. In our case, inhibition of murine DLL4 does not show any effect in mice xenografted with human PDTALL13 cells. We think that this discrepancy is due to the different experimental settings because we analyzed the overall survival, whereas in the previous work, Minuzzo et al, assessed only DLL4 role in early events, most probably associated with T-ALL cell colonization 4. Moreover, Minuzzo et al did not use mice xenografted with PDTALL13 cells, and this model might rely more on human DLL4 expression, and therefore might not be influenced by the antibody against the mouse ligand. Interestingly, Minuzzo et al detected DLL4 expression in spleen, already suggesting that DLL4 and T-ALL cell interaction is privileged in this organ 4. Notch plays a crucial role in PDTALL cell survival *in vitro* 4, 19. Similarly, both DBZ and demcizumab hampered PDTALL13 cell survival. Although demcizumab treatment strongly increased PDTALL13 cell death, at this moment we cannot rule out that Notch inhibition might affect also the cell cycle in these cells. Similarly, we do not know whether DLL4 drives T-ALL development in PDTALL13 or whether it is ectopically expressed later, but we clearly demonstrated that PDTALL13 cells are addicted to DLL4-Notch signaling.

In summary, we provided the proof of concept for the role of ectopic DLL4 expression in human T-ALL and also solid data indicating that spleen is required for tumor development in a mouse model of DLL4-driven T-ALL.

## Materials and Methods

### Mice

Tg8 and EF1a-N1IC mice were previously described 6, 16. NRG (NOD.Cg-Rag1tm1Mom Il2rgtm1Wjl/SzJ JAX^TM^) mice were purchased from Charles River (Margate, Kent, UK). All procedures used in this study were approved by the local Institutional and Animal Care Use Committee.

### Antibodies and staining

Thymocytes and splenocytes were homogenized, stained and data acquired with a BD FACSCalibur or BD LSRII apparatus. Data were analyzed with the FloJo software (BD Biosciences).

The following antibodies were used: anti-CD4 (clone H129.19, BD bioscience), anti-CD8 (53-6.7, Biolegend), anti-DLL4 monoclonal antibody YM152F (courtesy of Dr. M. Yan, Genentech; final staining concentration: 1 μg/mL), goat F(ab')2 anti-human Ig(gamma)-PE as secondary antibody (Invitrogen, H10104), and anti-NOTCH1 (HMN1-12, Biolegend). Other antibodies used were against Vβs [Vβ3 (JOVI.1, Caltag), 4 (KT4, Caltag), 5.1/5.2 (MR9-4, PharMingen), 6 (RR4-7, BD), 7(TR310, Caltag), 8.1/8.2 (MR5-2, BD), 8.3(1B3.3, PharMingen), 9 (MR10-2, BD), 12 (KT12, Caltag), 13 (mr12-3, BD) and 14 (14-2, PharMingen)], CD44 (1M7, eBioscience), CD25 (PC61.5, eBioscience), pTa (2F5, BD PharMingen), Thy1.2 (53-2.1, ebioscience), F4/80 (BM8, Biolegend), CD45 (Biolegend #103128; Clone: 30-F11), EpCam (Biolegend #118212; clone: G8.8), Ly51 (ebioscience #12-5995-81, clone: FG35.4), *Ulex europaeus* lectin (Vector Labs #FL-1061), CD80 (Biolegend #104721; clone: 16-10A1), and MHC class II (ebioscience #48-5321-82; clone: M5/114.15.2). For mouse immunohistochemistry, the following antibodies were used: anti-CD4 Alexa 647 (Biolegend #100531; clone: RM4-5), anti-CD8 Alexa 488 (Biolegend #100723; clone: 53-6.7), anti-CD4 615 (eBioscience #42-0042-80; clone: RM4-5), anti-MADCAM-1 (Biolegend #120705; clone: MECA-367), anti-c-Kit Alexa 488 (Biolegend #105815; clone: 2B8), anti-FcεRI (Biolegend #134301; clone: MAR-1), and anti-PNAd (Biolegend # 120803; clone: MECA-79). Secondary antibodies included: anti-rat IgG594 (Biolegend # 405410), goat anti-armenian hamster IgG Cy3 (Jackson Immunoresearch #127-0650160), goat anti-rat IgM Alexa Fluor 594 (Invitrogen #A21213), and goat anti-rat IgG Alexa 647 (Invitrogen #A-21247). For human cytometry analysis we used antibodies against: DLL4, clone MHD4-46, BioLegend #346507; CD45 clone HI30, eBioscience; 35-0459-42; and IgG-control clone MOPC-21, BioLegend, #400121.

### Ultrasound-guided intrathymic/intrasplenic injection

Four-week-old mice were anesthetized with 4% isoflurane (Aerrane, Baxter, Deerfield, IL) in medical air and maintained under anesthesia using a nose-cone with 1.5% isoflurane. Thymus or spleen was visualized with a 30-MHz 707B ultrasound probe (Visualsonics, Toronto, Canada), then 20 µl of a 5 mg/ml sulfo-NHS-biotin solution (Thermo Fisher Scientific) was injected in the thymus (10 µl in each lobe) using a Hamilton syringe and a 30G needle with the aid of a 3D micromanipulator. Similarly, 60-80 µl of 5 mg/ml sulfo-NHS-biotin solution was injected in the spleen in four spots.

### Splenectomy and thymectomy

For splenectomy, 3-week-old Tg8 mice were anesthetized with 0.6 ml/kg of a cocktail containing ketamine (12.5 mg/ml), xylazine (2.5 mg/ml) and acepromazine (0.5 mg/ml). An approximately 2.5 cm-long skin incision and a 1cm-long peritoneal wall incision were made using scissors on the left side of the body where the spleen lays. The spleen was gently pulled out. The artery and the afferent venules were tied off with a 4-0 suture. Then, spleen was cut and removed. The peritoneal wall and the skin were closed and sutured.

For thymectomy, 3-week-old Tg8 mice were similarly anesthetized and placed on a heat pad set at 37°C. A 1 cm ventral, horizontal skin incision centered over the clavicle was made, and the first 2-3 left ribs were cut near the sternum. The two thymic lobes were located and gently removed using forceps and vacuum aspiration. The chest cavity was compressed and the sutures were tightened to purge the air from the chest and reestablish negative pressure.

### Confocal microscopy

Tissues were embedded in optimum cutting temperature (OCT) compound and frozen at -80°C until use. Cryostat sections (8-10 mm-thick) were fixed with cold acetone at -20°C for 10 min and blocked in PBS with 5% BSA and 10% FCS at room temperature for 10 min. Confocal images were acquired with a Olympus Fluoview BX50W1 microscope and a Bio-Rad Radiance confocal system with 488-, 568- and 637- nm excitation lines, 560 and 650 nm detectors and HQ515/30 and HQ600/40 filters. Images were obtained with a Plan-Apochromat 20X (NA 0.8) and 60X (NA 1.4) immersion oil-objectives. Images were exported to Image J 1.43u for processing.

### Human sample analysis

Public RNA-seq gene expression data was obtained for human T-ALL samples 17. This gene expression data set contained FPKM values for 264 T-ALL samples, which were normalized using quantile normalization, and then transformed to corresponding TPM values. In order to identify a subset of samples with higher expression of DLL4 relative to the rest of samples, we log-transformed the TPM values for DLL4 to compute Z-scores and picked the samples that had Z > 2 to find observations that exceed the mean by at least 2 standard deviations. In order to determine if the samples with Z > 2 for DLL4 were similar to each other in terms of their gene expression profiles, we performed unsupervised hierarchical clustering of the samples from this data set using Pairwise Pearson Correlation Coefficient as the distance metric between the samples. For gene expression correlation we performed Pairwise Pearson correlation analysis.

### Protein expression in patient-derived T-ALL (PDTALL) and *in vitro* experiments

PDTALL were already developed before this study after informed consent from the patients and the approval by the University Medical Center Institutional Review Board committee 18. Spleens from NRG mice harboring the different PDTALL models were harvested and proteins extracted. Western blotting was performed as before 25 with anti-DLL4 (C19035, LSBio) and anti-GAPDH (#5174, Cell Signaling technologies) primary antibodies.

PDTALL9, 13 or 19 cells were harvested from the spleen of moribund NRG mice, resuspended and cultured in DMEM+20% FBS supplemented with non-essential amino acids and pyruvate. Cells were incubated with vehicle, murine anti-DLL4 (mDLL4), human anti-DLL4 (hDLL4) antibodies, or γ-secretase inhibitor (DBZ) for 24, 48 and 72 h with different concentrations for each treatment. At indicated timepoints cell viability was assessed by FACS using DAPI (Sigma) and an anti-human CD45 antibody to discriminate murine and human cells (clone HI30, #35-0459-42 eBioscience).

For apoptosis assay PDTALLs were expanded in mice and cultured as described above. Cells incubated with vehicle, murine anti-DLL4 or human anti-DLL4 (demcizumab) antibodies (both at 20 mg/ml), or γ-secretase inhibitor (DBZ, 500 nM) for 48h were analyzed by FACS (Cytoflex, Beckman Coulter) using an anti-human CD45 antibody (clone HI30, #35-0459-42, eBioscience), AnnexinV apoptosis detection kit (#556547, BD Pharmigen) and DAPI (Sigma).

### Patient-derived T-ALL (PDTALL) *in vivo* experiments

1×10^6^ PDTALL13 cells were IV injected in 32 irradiated NRG mice. Mice partial body irradiations were performed using a rotational X-ray irradiator (Xenx, Xstrahl, Experimental Radiotherapy Core Facility, IRCM, Montpellier, France). Single doses of 4.5 Gy were delivered at a dose rate of 2.7 Gy/min (225 kV and 13.6 mA) to the mice under sedation. After 1 week, mice were randomized (n = 8 mice/group) and treated with vehicle or anti-IgG control, 20 mg/kg/week of demcizumab (anti-human DLL4 antibody, Oncomed, Redwood City, CA, USA), 20 mg/kg/week of anti-mouse DLL4 antibody (Oncomed), or 3.3 mg/kg/day, from Monday to Thursday, of dibenzazepine (DBZ) (Synkon, Groningen, Netherlands), as previously described 26.

Spleens of moribund mice treated as above were formalin fixed and paraffin-embedded. For Immunofluorescence, the following antibodies were used: anti-HES1 (clone D6P2U, # 11988, Cell Signaling Technology); anti-human vimentine (vimentine (h); clone V9, # 790-2917), anti-mouse/human vimentine (vimentine (m/h); clone D21H3, #7541, Cell Signaling Technology) and DAPI (Sigma). All stainings were performed using Discovery Ultra automated system (Ventana) at RHEM (Réseau d'Histologie Expérimentale de Montpellier). Images were acquired using Thunder Imaging System (Leica) microscope. For each spleen, 4 random fields (×20 magnification) were analyzed using ImageJ software.

### Statistical Analysis

Unless otherwise specified, data are presented as the mean ± SEM. The Mann-Whitney test was used to assess the statistical significance of cytometry data. The Kaplan-Meier survival curves were analyzed with the Gehan-Breslow-Wilcoxon test. No mouse was excluded for the survival curves. Samples (cells or mice) were allocated to their experimental groups, according to their pre-determined type: cell type, mouse genotype, or mouse treatment. * *p* < 0.05; ** *p* < 0.01; *** *p* < 0.001; ****p < 0.0001.

## Supplementary Material

Supplementary figures.
