# Supplementary material for: Spleen plays a major role in DLL4-driven acute T-cell lymphoblastic leukemia
Source: Theranostics. 2021 Jan 1;11(4):1594–608. doi: 10.7150/thno.48067 (PMC7778594; doi:10.7150/thno.48067)
Supplement: Supplementary file 1 — Supplementary figures. [file thnov11p1594s1.pdf]

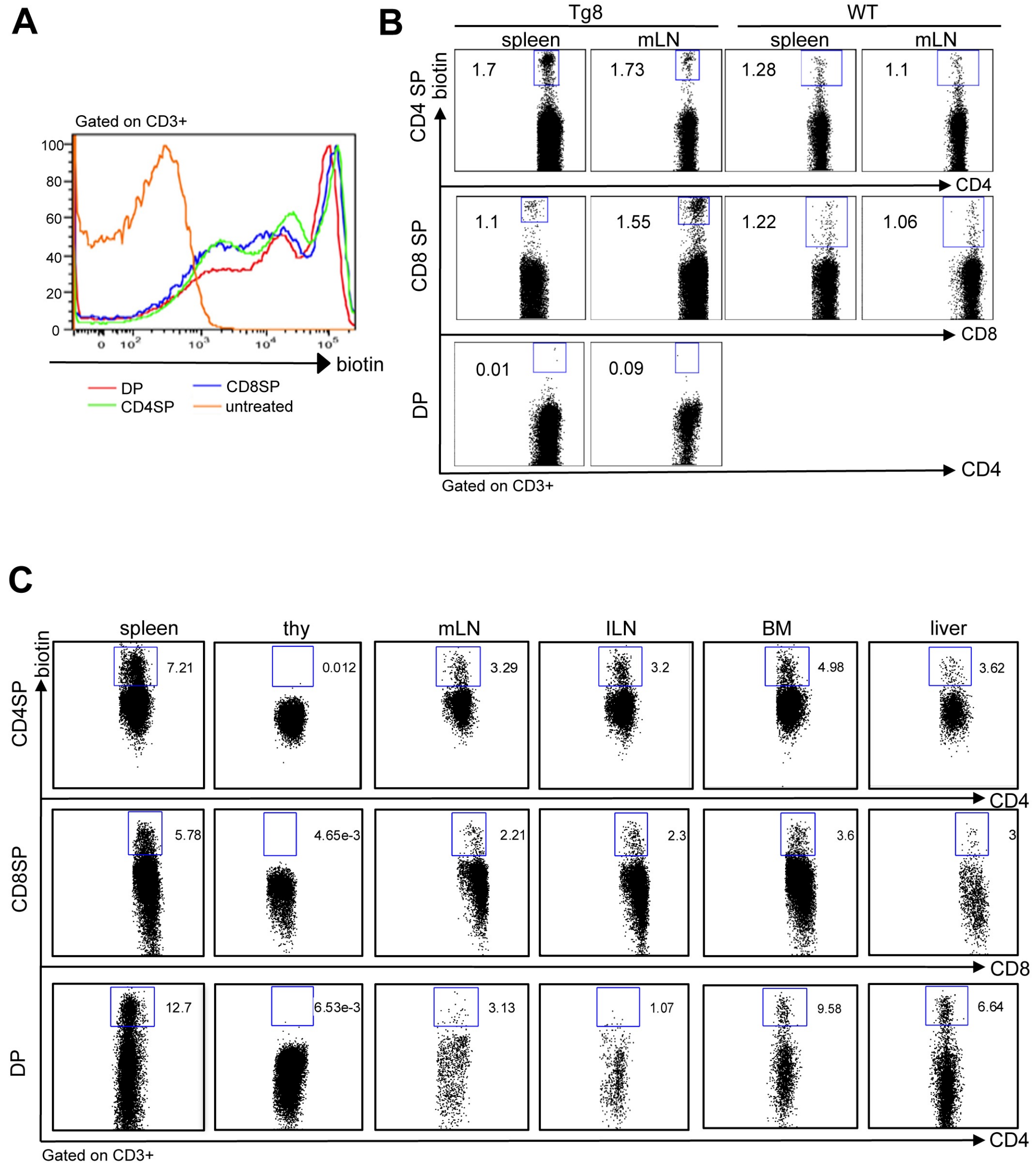

Figure S1

A) 24 h after intrathymic injection of biotin, mice were sacrificed and thymocytes were collected and stained with anti-CD4 anti-CD8 antibodies and streptavidin. Biotin labeling of CD4 single-positive (SP), double-positive (DP) and CD8SP cells is shown in the histogram. Tg8 mice that did not receive an intrathymic biotin injection were used as a negative control (untreated). Representative of n = 3 mice. Cells were positively gated on CD3 and then cells that expressed CD4 and/or CD8 were quantified for biotin. **B**) Biotin was injected in the thymus of 5-week-old

Tg8 and wild type (WT) mice. 24 h later, biotin-labeled cells emigrated from the thymus were identified in spleen and mesenteric lymph nodes (mLN) by staining with streptavidin. Representative image of n = 4 mice per genotype. Cells were positively gated on CD3 and then cells that expressed CD4 and/or CD8 were quantified for biotin. **C)** Biotin was injected in the spleen of 5-week-old Tg8 mice. 24 h later, biotin-labeled cells recently emigrated from spleen were identified in thymus, mLN, inguinal lymph nodes (ILN), bone marrow (BM), and liver. Representative image of n = 3 mice per genotype. Cells were positively gated on CD3 and then cells that expressed CD4 and/or CD8 were quantified for biotin.

CD4SP, $CD4^+$ single-positive cells; CD8SP, $CD8^+$ single-positive cells.

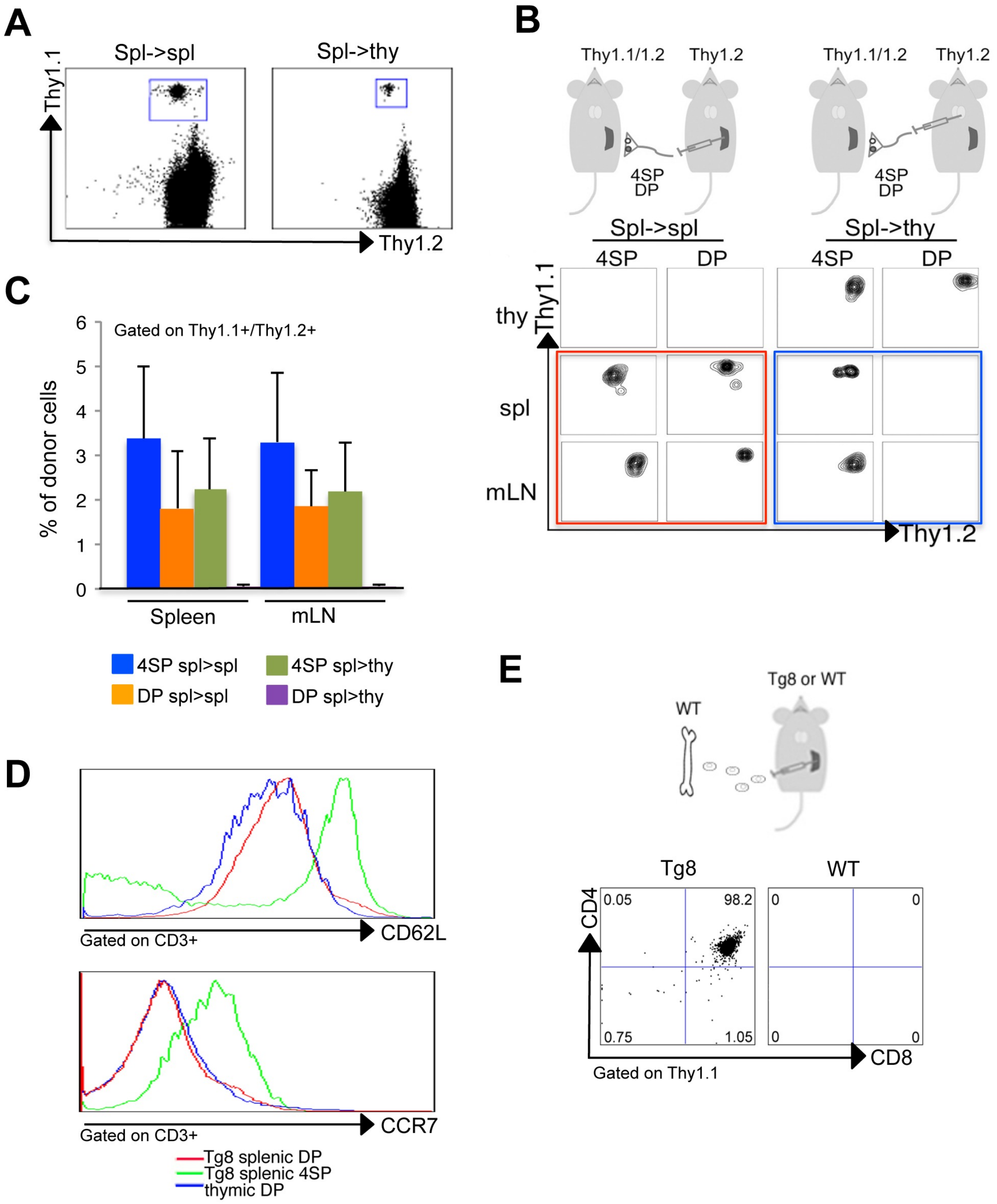

Figure S2

A) Example of gating strategy based in Thy1.1 and Thy1.2 staining patterns in the spleen>spleen and spleen>thymus conditions for (B) and (C) panels. **B**) Splenic DP cells and 4SP cells (as internal control) from Tg8 Thy1.1/1.2 donors were sorted and injected into spleen (left panel) or thymus (right panel) of Tg8 Thy1.2 recipients. Three days later cells were collected from thymus (thy), spleen (spl) and mesenteric lymph nodes (mLN) and stained with

anti-CD4, -CD8, -Thy1.1 and -Thy1.2 antibodies. Cells were positively gated on Thy1.1 and Thy1.2 double expression (as shown in A). Note that when injected into thymus, donor-derived DP cells were not found in spleen or mLN (the two rightmost plots), while they were found in mLN when injected into spleen (second lane from left, the plot at the bottom). Representative of n = 4 mice each group. **C**) Quantification of donor cells in each condition from the 4 mice in (B). **D**) Tg8 and WT thymocytes and splenocytes were re-suspended and stained with anti-CD62L and anti-CCR7 antibodies. Representative of n = 4 mice. Cells were positively gated on CD3 and then cells that expressed CD4 and/or CD8 were quantified for CD62L or CCR7. **E**) 2x10^{6} T cell-depleted bone marrow cells from 4-week-old WT Thy1.1 mice were purified and injected into the spleens of 4-week old Thy1.2 Tg8 and WT mice. 10 days later, the recipients' spleens were collected and stained for the Thy1.1 congenic marker to identify the donor-derived cells. Representative of n = 4 mice each genotype. Cells were positively gated on Thy1.1. and quantified for CD4 and CD8 expression.

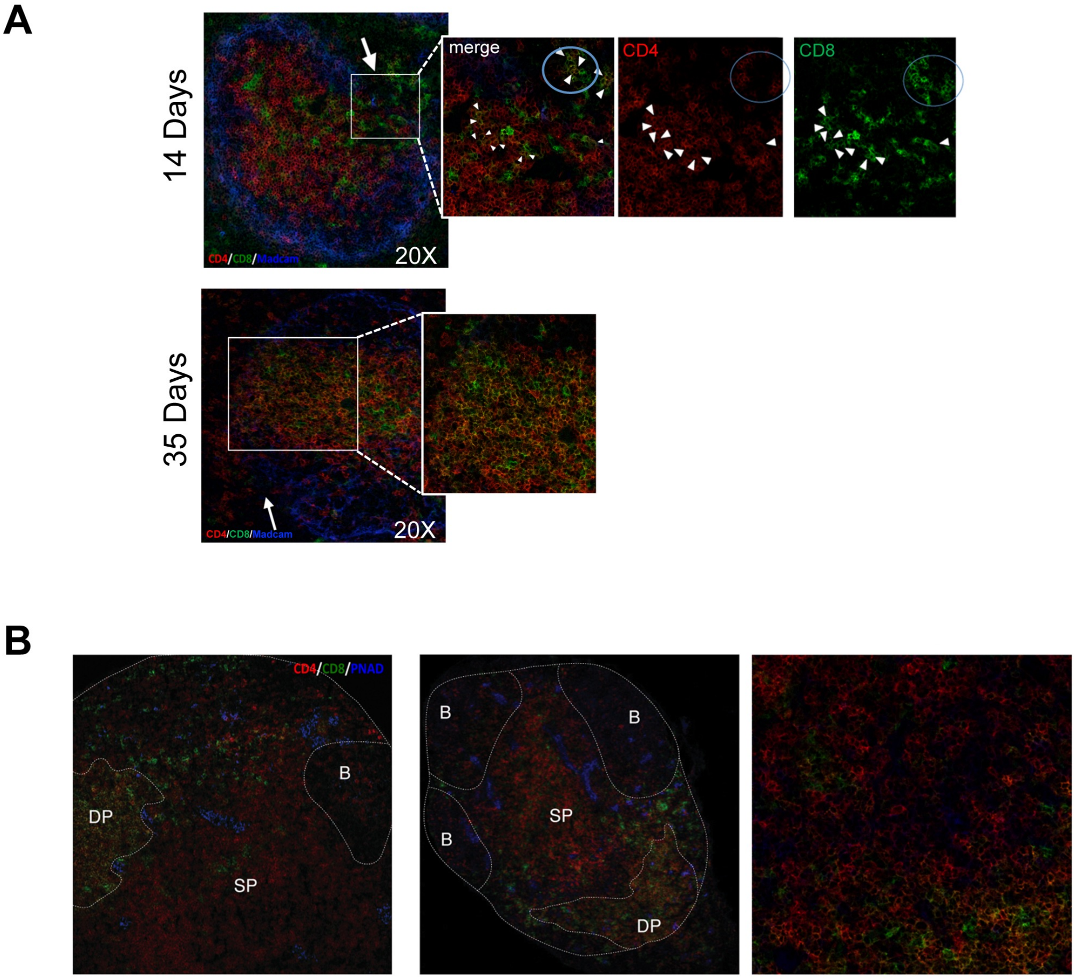

Figure S3

A) Spleens from 14- or 35-day old Tg8 mice were isolated, and prepared for frozen section analysis. Sections were stained with antibodies against CD4, CD8 and MAdCAM. Arrows indicate bridging channels (BC), which are the discontinuities of the marginal sinus endothelia. Arrowheads indicate $CD4^+CD8^+$ DP cells. The blue circle indicates PeriArteriolar Lymphoid Sheaths (PALS). Representative of n = 4 mice. **B**) Mesenteric lymph nodes of 35-day-old Tg8 mice were collected and prepared for frozen sections. Sections were fixed and incubated with anti-CD4, anti-CD8 and anti-PNA antibodies followed by secondary antibodies conjugated with different fluorescence dyes. Representative of n = 4 mice. SP, single positive; DP, double positive; and B, B cell area.

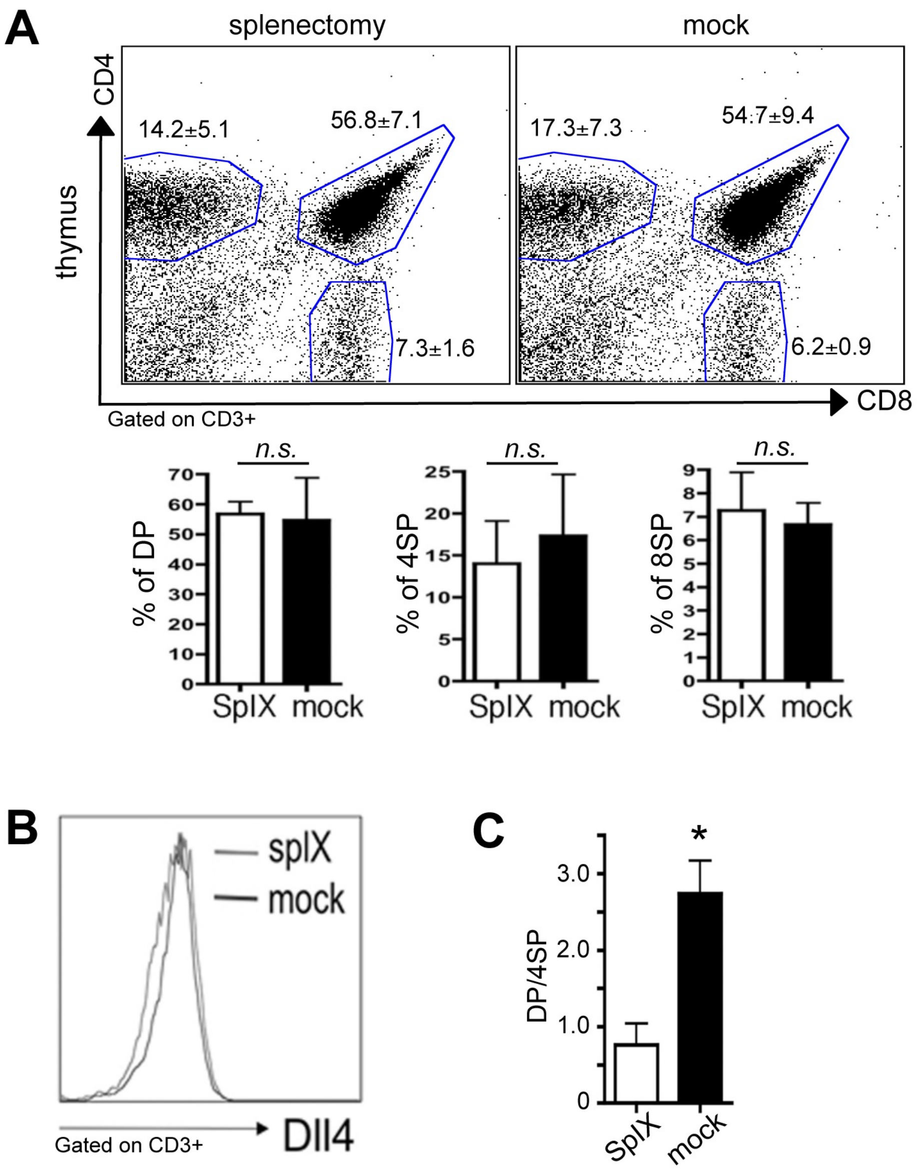

Figure S4

A) Representative cytometry images of CD4 and CD8 staining 2 months after splenectomy (splX) or mock surgery (mock). Percentages of DP, 4SP and 8SP cells in the thymus are shown. Data are the mean ± SEM, n = 4 mice per treatment. No significant difference between treatments was observed ($p > 0.05$, Mann–Whitney test). Cells were positively gated using CD3 expression, and then analyzed for CD4 and CD8 expression. **B**) CD4 single-positive (SP) cells in mLN were stained with an anti-DLL4 antibody. Representative data of n = 6 mice per treatment; SplX, splenectomy; mock, sham surgery. Cells were gated using CD3 and CD4 markers and analyzed

for DLL4 expression. **C**) $2x10^6$ bone marrow cells from EF1a-N1IC mice were injected intravenously in lethally irradiated WT congenic recipients. 4 weeks later, mice underwent splenectomy (SplX) or mock surgery (mock). Fourteen days after surgery, tamoxifen was injected intraperitoneally to induce NICD expression. At day 12 post-tamoxifen injection, blood cells were analyzed for CD4 and CD8 expression. Data are the mean ± SEM, n = 4 mice per genotype. Cells were positively gated using CD3 expression and then analyzed for CD4 and CD8 expression.
* $p < 0.05$ (Mann–Whitney test).

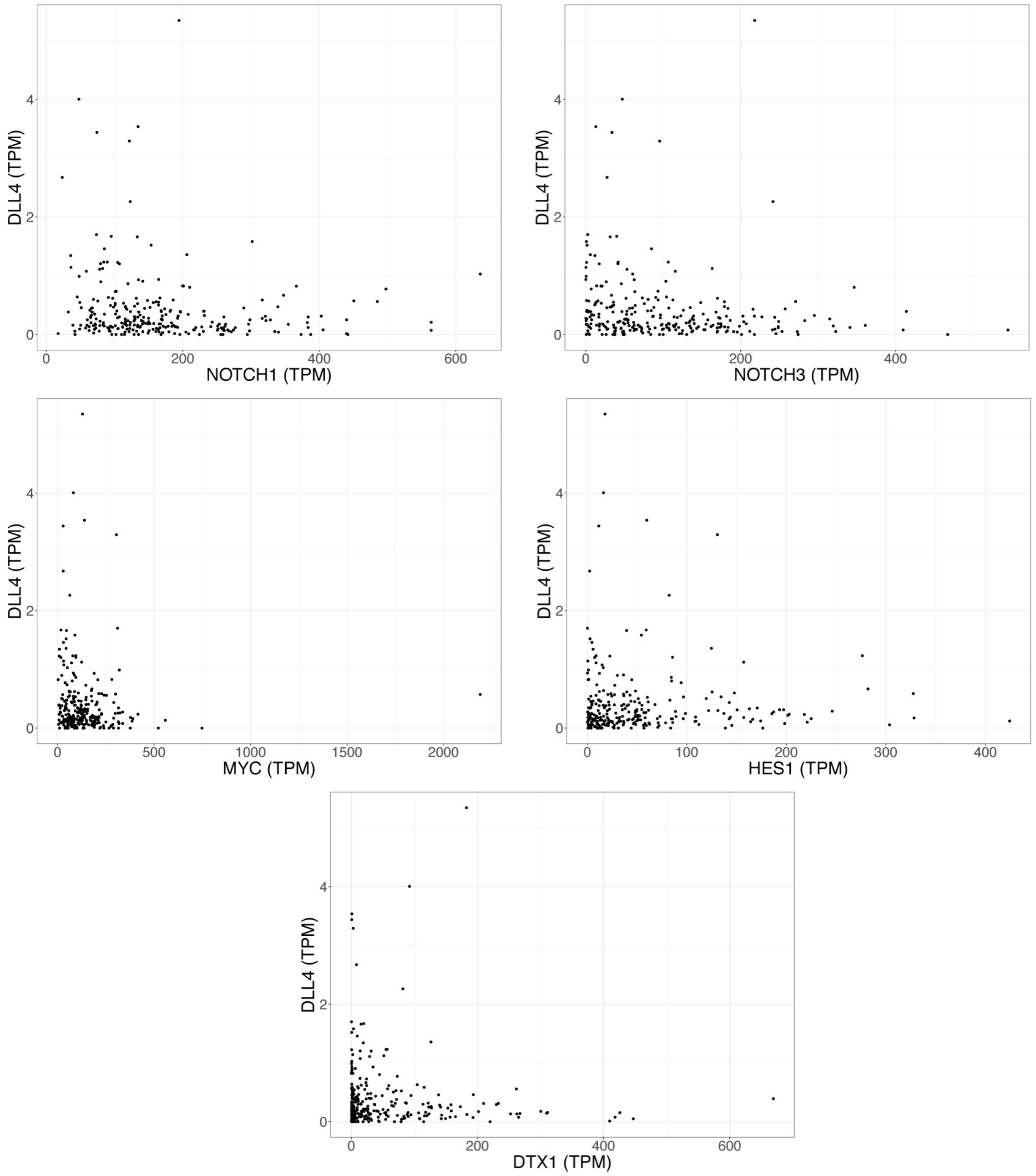

Figure S5.

Pairwise Pearson correlation test of *DLL4* and *NOTCH1*, *NOTCH3*, *MYC*, *HES1* and *DTX1* expression in the T-ALL dataset from Mullighan's laboratory. No positive correlation was found.

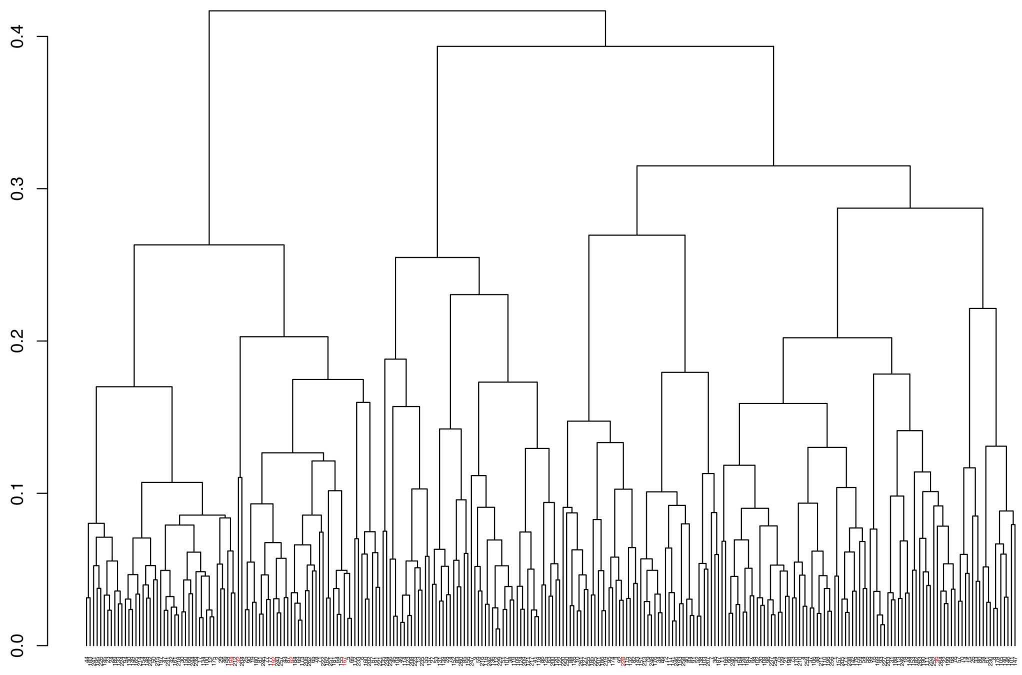

Figure S6.

Unsupervised clustering of the T-ALL samples in the dataset from Mullighan's laboratory using Pearson Pairwise correlation as the distance metric between samples. The analysis revealed that four of the seven DLL4-high expressing samples clustered together. In red all DLL4[high] T-ALL specimens.

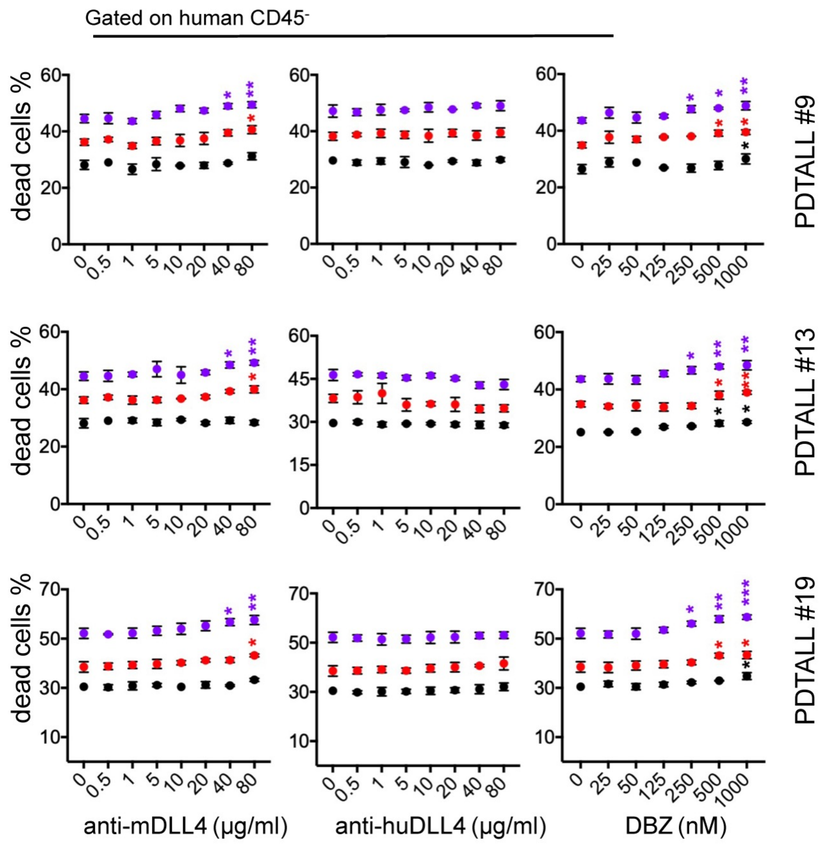

Figure S7.

PDTALL9, 13 and 19 cells were incubated with the indicated doses of murine anti-DLL4 antibody, human anti-DLL4 antibody (demcizumab), or DBZ for 1, 2 and 3 days, and then cell death was analyzed. Cells were negatively gated using a human anti-CD45 and analyzed for cell death using DAPI. Results for each day were compared to the control group (0) using two-way ANOVA (n = 2 per condition).

* $p < 0.05$, ** $p < 0.01$, *** $p < 0.001$.

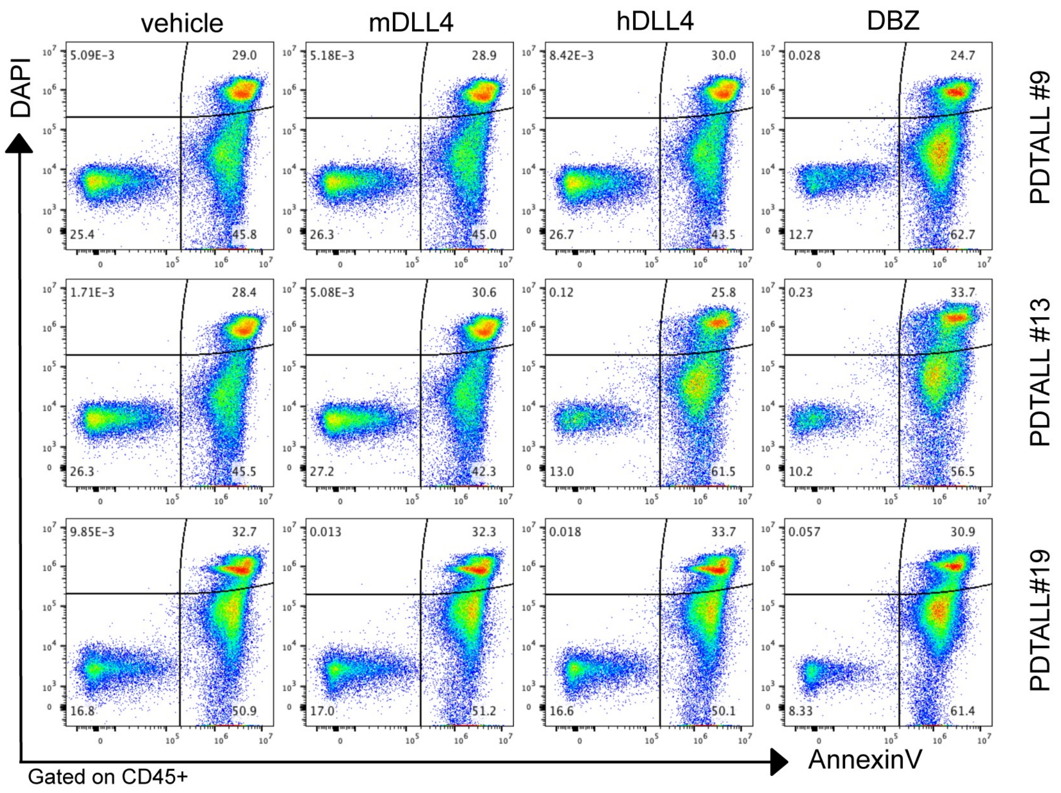

Figure S 8.

Apoptosis analysis performed by cytometry after AnnexinV and DAPI staining of the indicated PDTALLs that were gated positively using an anti-human CD45 antibody. Cells were incubated with 20 µg/ml for each antibody, 500 nM DBZ, or vehicle for 48 h. The treatment effect compared to control (vehicle) was assessed by two-way ANOVA (one example of each condition, n = 3 per condition).

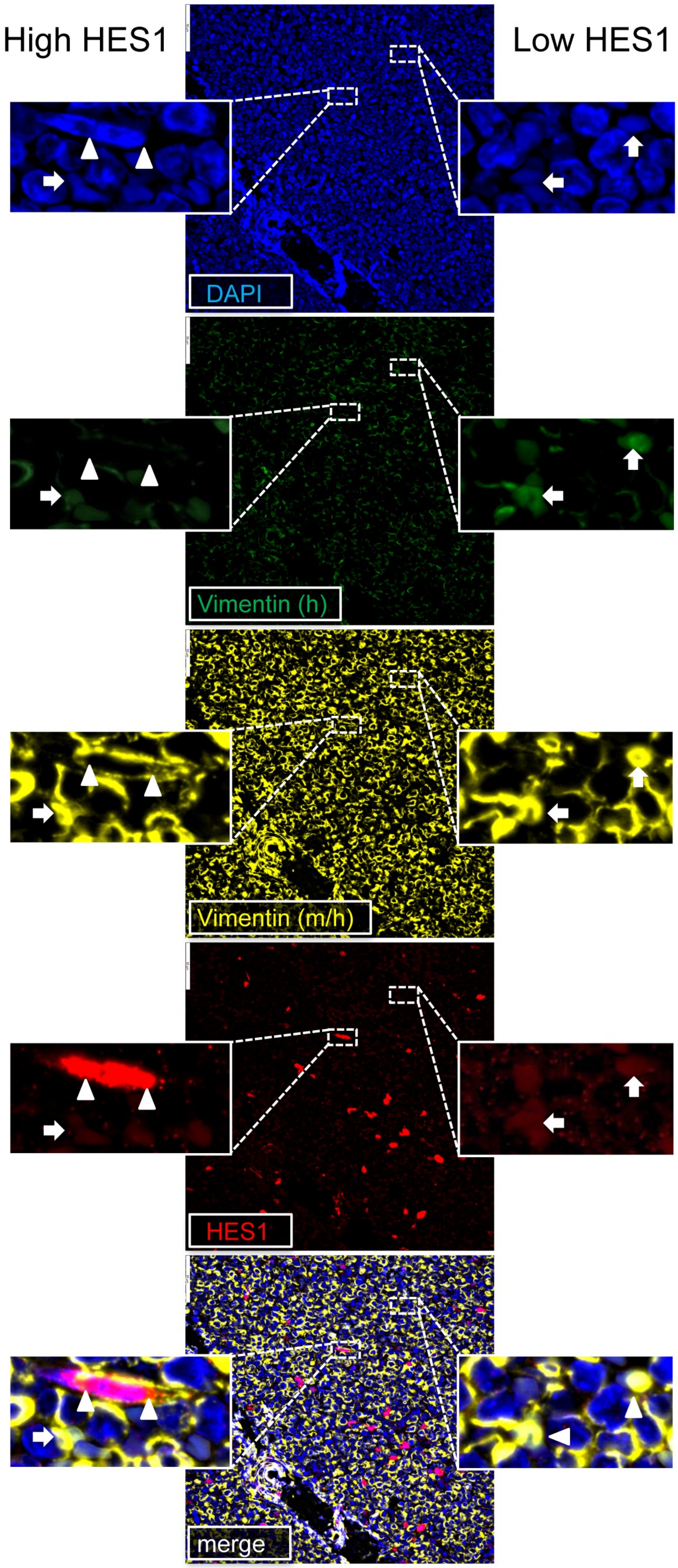
High HES1
Low HES1
DAPI
Vimentin (h)
Vimentin (m/h)
HES1
merge

Figure S9.

Formalin fixed–paraffin embedded spleen sections from NRG mice treated as in (Figure 6A) were incubated with anti-HES1, anti-vimentin (recognizing only human vimentin (h)), anti-vimentin (recognizing both human and mouse vimentin (m/h)), DAPI and secondary antibodies conjugated with fluorescent dyes.

Arrowheads indicates $HES1^{high}$ positive cells while arrows indicated $HES1^{low}$ positive cells. Scale bar 50 nm.
